# Genetic and virulence characteristics of hybrid Shiga toxin-producing and atypical enteropathogenic *Escherichia coli* strains isolated in South Korea

**DOI:** 10.3389/fmicb.2024.1398262

**Published:** 2024-05-15

**Authors:** Woojung Lee, Jina Ha, Jaehyun Choi, Yewon Jung, Eiseul Kim, Eun Sook An, Seung Hwan Kim, Hakdong Shin, Sangryeol Ryu, Soon Han Kim, Hae-Yeong Kim

**Affiliations:** ^1^Division of Food Microbiology, National Institute of Food and Drug Safety Evaluation, Ministry of Food and Drug Safety, Cheongju, Republic of Korea; ^2^Institute of Life Sciences & Resources and Department of Food Science and Biotechnology, Kyung Hee University, Yongin, Republic of Korea; ^3^Department of Food and Animal Biotechnology, Research Institute of Agriculture and Life Sciences, Seoul National University, Seoul, Republic of Korea; ^4^Department of Agricultural Biotechnology, Seoul National University, Seoul, Republic of Korea; ^5^Department of Biotechnology, and Carbohydrate Bioproduct Research Center, Sejong University, Seoul, Republic of Korea; ^6^Center for Food and Bioconvergence, Seoul National University, Seoul, Republic of Korea

**Keywords:** Shiga toxin-producing *E. coli*, enteropathogenic *E. coli*, whole genome sequencing, virulence factor, bacteriophages, cytotoxicity

## Abstract

**Introduction:**

The predominant hybrid pathogenic *E. coli*, enterohemorrhagic *E. coli* (EHEC), combines characteristics of Shiga toxin-producing *E. coli* (STEC) and enteropathogenic *E. coli* (EPEC), contributing to global outbreaks with severe symptoms including fatal consequences. Since EHEC infection was designated as a notifiable disease in 2000 in South Korea, around 2000 cases have been reported, averaging approximately 90 cases annually.

**Aim:**

In this work, genome-based characteristic analysis and cell-based assay of hybrid STEC/aEPEC strains isolated from livestock feces, animal source foods, and water in South Korea was performed.

**Methods:**

To identify the virulence and antimicrobial resistance genes, determining the phylogenetic position of hybrid STEC/aEPEC strains isolated in South Korea, a combination of real-time PCR and whole-genome sequencing (WGS) was used. Additionally, to assess the virulence of the hybrid strains and compare them with genomic characterization, we performed a cell cytotoxicity and invasion assays.

**Results:**

The hybrid STEC/aEPEC strains harbored *stx* and *eae* genes, encoding Shiga toxins and *E. coli* attachment/effacement related protein of STEC and EPEC, respectively. Furthermore, all hybrid strains harbored plasmid-carried enterohemolysin(*ehxCABD*), a key virulence factor in prevalent pathogenic *E. coli* infections, such as diarrheal disease and hemolytic-uremic syndrome (HUS). Genome-wide phylogenetic analysis revealed a close association between all hybrid strains and specific EPEC strains, suggesting the potential acquisition of Stx phages during STEC/aEPEC hybrid formation. Some hybrid strains showed cytotoxic activity against HeLa cells and invasive properties against epithelial cells. Notably, all STEC/aEPEC hybrids with sequence type (ST) 1,034 (*n* = 11) exhibited higher invasiveness than those with E2348/69. This highlights the importance of investigating potential correlations between STs and virulence characteristics of *E. coli* hybrid strains.

**Conclusion:**

Through genome-based characterization, we confirmed that the hybrid STEC/aEPEC strains are likely EPEC strains that have acquired STEC virulence genes via phage. Furthermore, our results emphasize the potential increased danger to humans posed by hybrid STEC/aEPEC strains isolated in South Korea, containing both *stx* and *eaeA*, compared to STEC or EPEC alone.

## 1 Introduction

*Escherichia coli* (*E. coli*) commonly resides in the gastrointestinal tracts of humans and animals as part of their natural gut flora (Kaper et al., 2004). Although most strains are harmless members of this gut community, certain *E. coli* variants, including those that have acquired specific virulence traits, can cause diarrheal and extraintestinal illnesses in humans and animals (Kaper et al., 2004; Dobrindt, 2005). Diarrheagenic *E. coli* (DEC) accounts for approximately 30–40% of acute diarrhea cases in children under 5 years old in developing countries (Dutta et al., 2013; Miliwebsky et al., 2016) and is a significant cause of both isolated instances and outbreaks of diarrhea worldwide (Croxen et al., 2013).

DEC, characterized by distinct combinations of virulence characteristics, are categorized into pathotypes, each with specific host preferences, global occurrence, disease impacts, and transmission methods (Croxen and Finlay, 2010). DEC are categorized into six main pathotypes: enterotoxigenic *E. coli* (ETEC), enteropathogenic *E. coli* (EPEC), Shiga toxin-producing *E. coli* (STEC), enteroinvasive *E. coli* (EIEC), enteroaggregative *E. coli* (EAEC), and diffusely adherent *E. coli* (DAEC). Each *E. coli* pathotype possesses distinct pathogenic mechanisms and a unique set of virulence factors encoded by specific gene clusters (Jesser and Levy, 2020; Pakbin et al., 2021). These genes related to pathogenicity may control functions such as adhesion, invasion, attachment, iron acquisition, motility, and toxin activity (Kaper et al., 2004; Croxen and Finlay, 2010).

STEC is an *E. coli* pathotype capable of producing at least one of the Shiga toxins (Stxs), namely Stx1 and Stx2 (Kaper et al., 2004; Croxen et al., 2013). Within each group, numerous subtypes or variants of Stx have been identified and characterized (Burgos and Beutin, 2012). STEC is a globally prevalent pathogen associated with human diseases that primarily causes diarrhea, hemorrhagic colitis (HC), and hemolytic-uremic syndrome (HUS) (Jesser and Levy, 2020; Pakbin et al., 2021). EPEC induces diarrhea and initiates the formation of attaching and effacing (A/E) lesions in the human gut epithelium (Kaper et al., 2004; Croxen et al., 2013). EPEC strains are categorized as either typical or atypical according to whether or not they have the *E. coli* adherence factor plasmid (EAF) expressing the *bfpA* gene. This gene is responsible for the production of bundle-forming pili. Typical EPEC (tEPEC) possesses both the locus of enterocyte effacement (LEE) region responsible for attaching and causing effacing lesions (*eae*), and *bfpA*. In contrast, atypical EPEC (aEPEC) lacks *bfpA* (Jesser and Levy, 2020; Pakbin et al., 2021). aEPEC strains exhibit comparable prevalence rates among healthy adults and children with diarrhea, and are also found in asymptomatic adults (Hazen et al., 2016). However, certain aEPEC strains have been linked to diarrhea outbreaks (Vieira et al., 2016).

The Enterohemorrhagic *Escherichia coli* (EHEC) is commonly used to refer to a subset of STEC strains that simultaneously harbors genes for Stx production and LEE. As STEC strains have the potential to induce diarrhea and hemolytic uremic syndrome (HUS) in human subjects regardless of the presence of LEE, this PAI is an accessory set of virulence genes that enhances STEC pathogenicity. EHEC are considered a pathotype of *E. coli* and are referred to as such in the scientific literature rather than referring to them as hybrid STEC/aEPEC. However, according to previous studies, there are reports indicating that EPEC/STEC hetero-pathogens consist of atypical EPEC carrying Stx (Croxen et al., 2013; Eichhorn et al., 2015; Gomes et al., 2016; Silva et al., 2019). Especially, Gioia-Di Chiacchio et al. (2018) were the first to report a typical EPEC strain carrying Stx, identified in eight *E. coli* strains isolated from birds, which simultaneously harbored Stx2, LEE, and BFP.

Globally, emerging hybrid diarrheagenic *E. coli* strains incorporating genetic markers from diverse pathotypes pose substantial public health concerns (Santos et al., 2020). This emergence is attributed to the horizontal gene transfer (HGT) among diarrheagenic *E. coli* or the prevalence of *E. coli* virulence genes typically located on plasmids, enabling transmission via conjugation (Kaper et al., 2004; Dobrindt, 2005; Geurtsen et al., 2022). Hybrid DEC pathotype strains are increasingly reported worldwide. Different hybrid strains, such as EPEC/ETEC, have been identified in India (Dutta et al., 2015), with Germany (Bielaszewska et al., 2011) and Indonesia (Setyarini et al., 2020) reporting STEC/EAEC hybrids. Brazil has documented cases of EPEC/STEC (Gioia-Di Chiacchio et al., 2018) and EPEC/EAEC (de Lira et al., 2021). Sweden has several instances of STEC/ETEC (Bai et al., 2019). Diarrheal disease outbreaks associated with various DEC pathotypes occurred in Germany in 2011 (Buchholz et al., 2011), Japan in 2016 (Furukawa et al., 2018) and 2020 (Kashima et al., 2021), Nottingham, UK in 2014 (Newitt et al., 2016), South Korea in 2018 (Lim et al., 2020), and the United Kingdom in 2020 (Mulchandani et al., 2021). Hybrid strains are characterized by the presence of virulence genes unique to multiple *E. coli* pathotypes, typically detected via PCR or comprehensive whole-genome sequencing (WGS) (Jesser and Levy, 2020; Lee et al., 2023a,b). Cell-based assays have also been used to assess the virulence potential of hybrid strains (Valiatti et al., 2020).

The purpose of the present study was to characterize the genetic structure and evaluate the virulence potential of hybrid STEC/aEPEC strains isolated from livestock feces, animal source foods, and water in South Korea. This study enhances our understanding of the horizontal transfer of several virulence-related genes via mobile genetic elements (MGEs) and provides information on the virulence potential of hybrid STEC/aEPEC strains. Based on our findings, we highlighted the potential implications of these hybrid *E. coli* strains for public health, emphasizing the need for comprehensive monitoring to mitigate risks associated with the horizontal transfer of virulence-related genes.

## 2 Materials and methods

### 2.1 Bacterial strains and serotyping

Pathogenic *E. coli* strains (*n* = 1,433) were isolated from animal food (beef, pork, chicken, and duck) (*n* = 961), animal feces (cattle, pigs, and poultry) (*n* = 358), vegetables such as salad (*n* = 75), and other (*n* = 39) during 2015 to 2021 in the Korea and distributed by the Korean Culture Collection for Foodborne Pathogens (Ministry of Food and Drug Safety, Cheongju, Republic of Korea). The 32 hybrid strains used in this study are summarized in Table 1. The strains were identified using a VITEK MS system (BioMérieux Inc., Marcy-l’Etoile, France). Additionally, to identify the serotype, bacteria were tested for agglutination using specific somatic (O1–O181) antisera from the *E. coli* Reference Laboratory (LREC) in Lugo, Spain. This method helps detect variations in somatic (O) antigens (Guinée et al., 1972, 1981; Blanco et al., 1996).

**Table 1:** Summarized characteristics of hybrid STEC/aEPEC strains.

Strain name	Collection date	Geographic location	Isolation source
MFDS1006809	Jun 9, 2015	Jeollabuk-do	Drinking water
MFDS1008621	Mar 13, 2017	Jeollabuk-do	animal source foods (beef)
MFDS1008630	May 15, 2017	Chungcheongbuk-do	animal source foods (beef)
MFDS1008632	May 15, 2017	Gyeonggi-do	animal source foods (beef)
MFDS1008635	May 22, 2017	Jeollabuk-do	animal source foods (beef)
MFDS1009661	Aug 7, 2017	Jeollabuk-do	animal source foods (pork)
MFDS1010542	Aug 22, 2017	Chungcheongbuk-do	animal source foods (beef)
MFDS1011019	Mar 26, 2018	Gyeonggi-do	animal source foods (beef)
MFDS1011030	May 1, 2018	Gyeonggi-do	animal source foods (beef)
MFDS1011031	May 1, 2018	Chungcheongbuk-do	animal source foods (beef)
MFDS1011035	May 14, 2018	Jeollabuk-do	animal source foods (beef)
MFDS1011124	Jun 4, 2018	Jeollanam-do	animal source foods (beef)
MFDS1012302	Jul 24, 2018	Gyeonggi-do	animal source foods (pork)
MFDS1012330	Oct 15, 2018	Gyeonggi-do	animal source foods (beef)
MFDS1013792	Feb 28, 2019	Incheon	livestock feces (cattle)
MFDS1014197	Dec 21, 2019	Jeollabuk-do	livestock feces (cattle)
MFDS1014198	Dec 21, 2019	Jeollabuk-do	livestock feces (cattle)
MFDS1014199	Dec 21, 2019	Jeollabuk-do	livestock feces (cattle)
MFDS1016241	Feb 10, 2020	Chungcheongnam-do	livestock feces (cattle)
MFDS1017043	May 11, 2020	Gyeonggi-do	animal source foods (beef)
MFDS1017062	Jun 8, 2020	Chungcheongbuk-do	animal source foods (beef)
MFDS1017065	Jun 8, 2020	Chungcheongbuk-do	animal source foods (beef)
MFDS1018250	Sep 18, 2020	Daejeon	livestock feces (cattle)
MFDS1019512	May 4, 2021	Chungcheongnam-do	livestock feces (cattle)
MFDS1019513	May 4, 2021	Gyeonggi-do	livestock feces (cattle)
MFDS1019524	Jun 8, 2021	Chungcheongbuk-do	livestock feces (cattle)
MFDS1020945	Jun 28, 2021	Gyeonggi-do	animal source foods (beef)
MFDS1020986	Jun 7, 2021	Chungcheongbuk-do	animal source foods (beef)
MFDS1021098	May 17, 2021	Jeollanam-do	animal source foods (beef)
MFDS1021101	Jun 21, 2021	Chungcheongbuk-do	animal source foods (beef)
MFDS1021102	Jun 21, 2021	Jeollanam-do	animal source foods (beef)
MFDS1022661	Jun 28, 2021	Gyeonggi-do	animal source foods (beef)

### 2.2 Real-time PCR-based identification of hybrid strains

Real-time PCR was performed using a PowerCheckTM 20/15 Pathogen Multiplex Real-time PCR kit (Kogen Biotech Co., Ltd., Seoul, Republic of Korea) to detect virulence genes in different DEC pathogens, including *VT1* and *VT2* (STEC); *bfpA* and *eaeA* (EPEC); *LT*, *STh* and *STp* (ETEC); *aggR* (EAEC); *ipaH* (EIEC) (Lee et al., 2023a,b).

### 2.3 Genome sequencing, assembly, and annotation

Genomic DNA was extracted using the MagListo™ 5 M Genomic DNA Extraction Kit for cells and tissues (Bioneer, Daejeon, Korea) following the manufacturer’s instructions. A DNA library was prepared using the Nextera DNA Flex Library Prep Kit (Illumina, San Diego, CA, USA). Short-read sequencing was performed using the Illumina MiSeq sequencing system with the MiSeq Reagent Kit v3 (600-cycle). A hybrid genome assembly approach was employed to ensure a comprehensive genome sequence by integrating additional long-read sequence data obtained from PacBio Sequel (Pacific Bioscience, Menlo Park, CA, USA) or ONT MinION (Oxford Nanopore Technologies, Oxford, UK). A hybrid assembly of short-read sequence data (Illumina MiSeq) and long-read sequence data (PacBio Sequel, ONT MinION) was performed using Unicycler (v0.4.9^1^; default options). The assembled genome was annotated using the Rapid Annotation with the Subsystem Technology toolkit, implemented in the BV-BRC annotation web service (v3.32.13a).

### 2.4 DNA sequence analysis and bioinformatics

Antimicrobial resistance genes and virulence factors were identified using ResFinder v4.1 and the virulence factor database (Bortolaia et al., 2020; Liu et al., 2022). Pathogenic and toxigenic factors essential for EPEC pathogenesis in the human intestine, including LEE-encoded virulence factors, were identified using the Basic Local Alignment Search Tool (BLAST). RM10042 and RM10466 were used as reference strains for STEC (Parker et al., 2018). And E2348/69 and E110019 were used as reference strains for EPEC (Iguchi et al., 2009; Hazen and Rasko, 2019). Genome data for each strain were acquired from the NCBI database, with the corresponding accession numbers as follows: RM10042 (CP028122), RM10466 (CP028381), E2348/69 (FM180568), and E110019 (CP035751). MGEs were identified using MobileElementFinder^2^ (Camacho et al., 2009). Whole genome multi-locus sequence typing (wgMLST) was performed using BioNumerics (v8.0; Applied Maths, Sint-Martens-Latem, Belgium), and the analysis included 17,380 loci from *E. coli* and *Shigella*. Additionally, SerotypeFinder 2.0 (Joensen et al., 2015), a web-based serotyping tool, was used to predict the antigen profiles of different *E. coli* strains.

### 2.5 Prophage prediction and analysis

The PHAge Search Tool Enhanced Release (PHASTER) (Arndt et al., 2016) was used to detect bacteriophage sequences within distinct genome sequences. PHASTER predicted potential prophage regions, classifying them as “intact (scoring above 90),” “questionable (with scores ranging between 70 and 90),” or “incomplete (scoring below 70),” based on the presence of phage-related genes in the identified phage region of the genome containing Stx. Following the identification of these prophage sequences, the RAST toolkit within the BV-BRC genome annotation web service (v3.32.13a) was used for the annotation of virulence genes.

### 2.6 Identification of plasmid-associated sequences

PlasmidFinder 2.1 was used to investigate the plasmid features of the entire genome (Carattoli et al., 2014). The threshold for identification was 95% and the minimum coverage was set at 60%. Identification was based on the discovery of replicon sequences from various known plasmid incompatibility (Inc) groups, forming the basis for the identification process. Extracted plasmid sequences were annotated to detect virulence genes, including hemolysin-related genes, using Rapid Annotation with Subsystem Technology tools in the BV-BRC annotation web service (v3.32.13a).

### 2.7 Phylogenetic analysis and population structure analysis

We conducted a comparative genomic analysis of 32 hybrid STEC/aEPEC strains isolated in South Korea and 187 other pathogenic *E. coli* strains (Lee et al., 2023a,b). To analyze the pan-genome, we used the bacterial pan-genome analysis (BPGA) tool (v1.3) with default parameters. Clustering was performed using the USEARCH tool with 95% sequence identity as the cutoff value. A phylogenetic tree was constructed using the neighbor-joining method and visualized using the Interactive Tree of Life (iTOL) v6. For population structure analysis, RhierBAPs (Tonkin-Hill et al., 2018) were used.

### 2.8 Cell culture

HeLa cells (ATCC CCL-2) were grown in Dulbecco’s modified Eagle’s medium (Gibco, Thermo Fisher Scientific, Inc., Waltham, MA, USA) supplemented with 10% fetal bovine serum (Gibco) and incubated at 37°C for 24 h in a 5% CO_2_ environment. Cell monolayers were seeded in 96-well tissue culture plates at a density of 5 × 10^4^ cells/well for the cytotoxicity assay and 1 × 10^5^ cells/well for the invasion assay (Horwitz and Latimer, 2000; Shifrin et al., 2002.).

### 2.9 Cell cytotoxicity and invasion assays

For cytotoxicity assessment, hybrid STEC/aEPEC strains were prepared as previously described (Roberts et al., 2001). The supernatants from 10^7^ CFU of polymyxin B-treated bacteria were obtained and applied to the cell monolayer. Cell cytotoxicity was evaluated by measuring lactate dehydrogenase (LDH) release using the cytotoxicity detection kit (LDH) (Roche, Basel, Switzerland) according to the manufacturer’s instructions. The absorbance was measured at 495 nm using a SpectraMax i3 platform (Molecular Devices, CA, USA). The results were calculated as relative values compared to those of untreated cells. The LDH release (% cytotoxicity) was calculated according to the following equation: ((LDH_treated_ – LDH_untreated_)/(LDH_totallysis_ − LDH_untreated_)) × 100. The experiments were performed in triplicate in duplicate wells. *E. coli* DH5α and the STEC strain ATCC 43890 were used as the non-cytotoxic control and cytotoxic positive control in the cytotoxicity assessment (Lang et al., 1994; Robinson et al., 2006).

To assess the invasive abilities of hybrid strains, a classical gentamicin protection assay was performed as previously described (Choi et al., 2007), with minor modifications. The cultures of hybrid STEC/aEPEC strains were applied to the cell monolayer at a multiplicity of infection (MOI) of 100. Invasion ability was determined as the percentage of the initial colony-forming units recovered after gentamicin treatment and expressed as relative invasion (%) of the non-invasive control strain, *E. coli* DH5α, defined as 100%. The experiments were performed twice in duplicate wells. *E. coli* DH5α and the EPEC strain E2348/69 were used as the non-invasive control and invasive positive control in the cell invasion assay (Rosa et al., 2001; Robinson et al., 2006; Iguchi et al., 2009).

### 2.10 Statistical analysis

The significance of differences between the bacterial strains was assessed using the GraphPad Prism 5.01 software, by using the Student’s unpaired *t*-test. The data are represented as means and standard errors of the mean (SEM) from two independent experiments.

## 3 Results

### 3.1 Serotyping and sequence type of STEC/aEPEC hybrids

*E. coli* O and H antigens were identified using specific somatic (O1–O181) antisera and with the sequence based SerotypeFinder 2.0. The sequence type (ST) was determined using the wgMLST database for *E. coli* and *Shigella* species. Serotyping and sequencing results for the 32 hybrid STEC/aEPEC strains are summarized in Table 2. A comparison between *in vitro* and *in silico* serotyping showed consistent findings across the 32 hybrid STEC/aEPEC strains. Based on *in vitro* and *in silico* serotyping, the 32 hybrid *E. coli* strains belonged to 10 distinct O:H serogroups [O103:H2 (*n* = 13), O26:H11 (*n* = 3), O74:H25 (*n* = 3), O84:H2 (*n* = 3), O108:H25 (*n* = 3), O98:H21 (*n* = 2), O103:H8 (*n* = 2), O71:H8 (*n* = 1), O156:H25 (*n* = 1), and O177:H25 (*n* = 1)]. The hybrid strains represented seven STs [ST1034 (*n* = 11), ST591 (*n* = 5), ST774 (*n* = 5), ST481 (*n* = 4), ST1249 (*n* = 2), ST1621 (*n* = 2), ST2366 (*n* = 2), and ST2277 (*n* = 1)].

**Table 2:** Genomic characteristics on hybrid STEC/aEPEC strains.

Strain name	Size (bp)	GC (%)	CDSs	Accession no.	Plasmids	Serotype		Sequence type	*stx* subtype
MFDS1006809	5,604,335	50.7	5,786	JAVRGG000000000	1	O103	H2	ST1034	*stx1a*
MFDS1008621	5,314,960	50.6	5,518	JAVRGH000000000	2	O108	H25	ST591	*stx1a*
MFDS1008630	5,414,609	50.7	5,788	JAVRGI000000000	2	O103	H2	ST1034	*stx1a*
MFDS1008632	5,544,494	50.7	5,751	JAVRGJ000000000	2	O103	H2	ST1034	*stx1a*
MFDS1008635	5,902,706	50.7	6,414	JAVRGK000000000	1	O26	H11	ST481	*stx1a*
MFDS1009661	5,591,527	50.7	5,890	JAVQNV000000000	2	O71	H8	ST481	*stx1a, stx2a, stx2c*
MFDS1010542	5,939,439	50.7	6,353	JAVRGL000000000	2	O103	H2	ST1034	*stx1a*
MFDS1011019	5,388,644	50.5	5,563	JAVRGM000000000	3	O108	H25	ST591	*stx1a*
MFDS1011030	5,984,701	50.7	6,381	JAVRGN000000000	2	O26	H11	ST481	*stx1a*
MFDS1011031	5,639,633	50.7	5,815	JAVRGO000000000	2	O103	H2	ST1034	*stx1a*
MFDS1011035	5,694,797	50.7	5,894	JAVRGP000000000	1	O103	H2	ST1034	*stx1a*
MFDS1011124	5,322,822	50.6	5,437	JAVRGQ000000000	1	O108	H25	ST591	*stx1a*
MFDS1012302	5,491,618	50.6	5,757	JAVRGR000000000	2	O156	H25	ST591	*stx1a*
MFDS1012330	5,674,314	50.7	5,901	JAVRGS000000000	1	O103	H2	ST1034	*stx1a*
MFDS1013792	5,681,786	50.7	5,982	JAVRGT000000000	1	O98	H21	ST774	*stx1a*
MFDS1014197	5,558,649	50.6	5,755	JAVRGV000000000	1	O98	H21	ST774	*stx1a*
MFDS1014198	5,430,889	50.6	5,556	JAVRGW000000000	1	O84	H2	ST774	*stx1a*
MFDS1014199	5,429,145	50.6	5,566	JAVRGX000000000	1	O84	H2	ST774	*stx1a*
MFDS1016241	5,549,023	50.6	5,860	JAVRGZ000000000	3	O74	H25	ST591	*stx1a*
MFDS1017043	5,560,371	50.7	5,723	JAVRHA000000000	1	O103	H2	ST1034	*stx1a*
MFDS1017062	5,647,287	50.7	5,894	JAVRHB000000000	2	O103	H2	ST1034	*stx1a*
MFDS1017065	5,455,814	50.8	5,732	JAVRHC000000000	1	O103	H2	ST1034	*stx1a*
MFDS1018250	5,883,095	50.5	6,183	JAVRHD000000000	2	O26	H11	ST481	*stx1a*
MFDS1019512	5,510,506	50.6	5,882	JAVQNW000000000	3	O74	H25	ST1621	*stx1a*
MFDS1019513	5,510,470	50.6	5,857	JAVQNX000000000	3	O74	H25	ST1621	*stx1a*
MFDS1019524	5,379,618	50.5	5,794	JAVQNY000000000	2	O177	H25	ST2277	*stx1a, stx2c*
MFDS1020945	5,532,440	50.6	8,992	JAVQNZ000000000	1	O103	H8	ST2366	*stx1a*
MFDS1020986	5,967,194	50.3	9,866	JAVQOA000000000	2	O84	H2	ST774	*stx1a*
MFDS1021098	5,497,770	50.5	8,896	JAVQOB000000000	1	O103	H2	ST1034	*stx1a*
MFDS1021101	5,643,415	50.6	9,281	JAVQOC000000000	1	O103	H2	ST1249	*stx1a*
MFDS1021102	5,642,375	50.6	9,388	JAVQOD000000000	1	O103	H2	ST1249	*stx1a*
MFDS1022661	5,537,305	50.7	5,823	JAVQOE000000000	1	O103	H8	ST2366	*stx1a*

CDS, coding DNA sequence.

### 3.2 Genome assemblies of STEC/aEPEC hybrids

Thirty-two hybrid STEC/aEPEC strains from South Korea were sequenced; 16 strains had one chromosome and one plasmid, 11 had one chromosome and two plasmids, and five had one chromosome and three plasmids. Genomic characteristics of the hybrid STEC/aEPEC strains are summarized in Table 2. The genome lengths of these strains ranged from 5,314,960 to 5,984,701 bp. In addition, these strains had G + C contents between 50.3 and 50.8%, the number of coding DNA sequences was between 5,437 and 9,866, tRNA genes between 87 and 105, and rRNA genes between 22 and 26.

### 3.3 *In silico* identification of virulence genes

**Figure 1:**
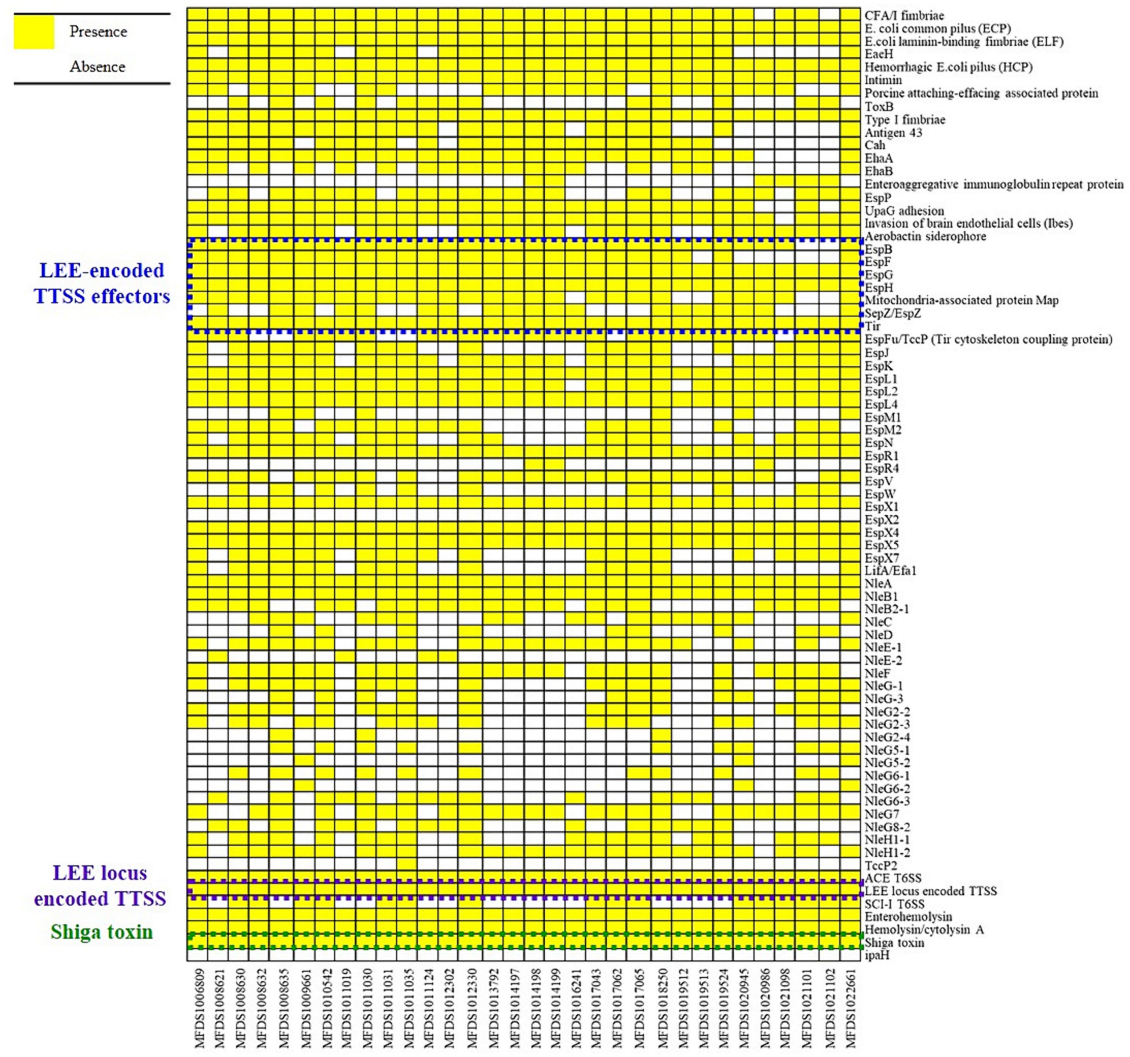
Heatmap of virulence factors across hybrid STEC/aEPEC strains. Heatmap showing the presence/absence of virulence factors (y-axis) within hybrid STEC/aEPEC isolates identified in this study (x-axis). The presence of virulence genes is shown in yellow, and the absence in white. Heatmap produced using the gplot (v3.1.3) package in R (v4.1.3).

**Figure 2:**
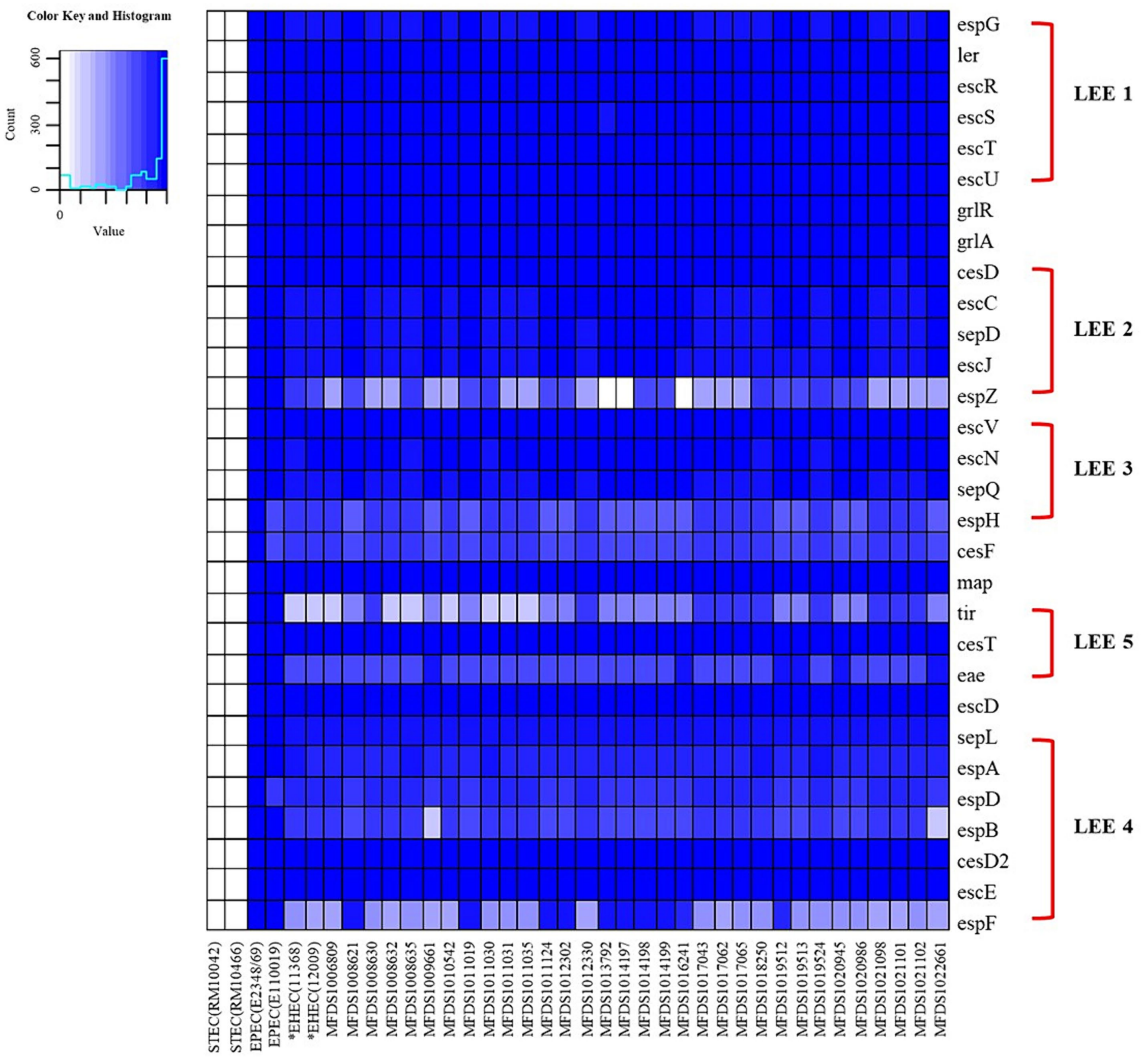
The heatmap of the protein-encoding genes within the LEE region of STEC strains (RM10042 and RM10466), EPEC strains (E2348/69 and E110019), non-O157 EHEC strain (11,368 and 12,009), and STEC/EPEC genomes analyzed in this study. Heatmap showing the presence/absence of protein-encoding genes (y-axis) within hybrid STEC/aEPEC isolates identified in this study (x-axis). The presence of protein-encoding genes within the LEE region is shown in blue, whereas the absence is shown in white, as indicated in the color key. Heatmap produced using the gplot (v3.1.3) package in R (v4.1.3).

**Figure 3:**
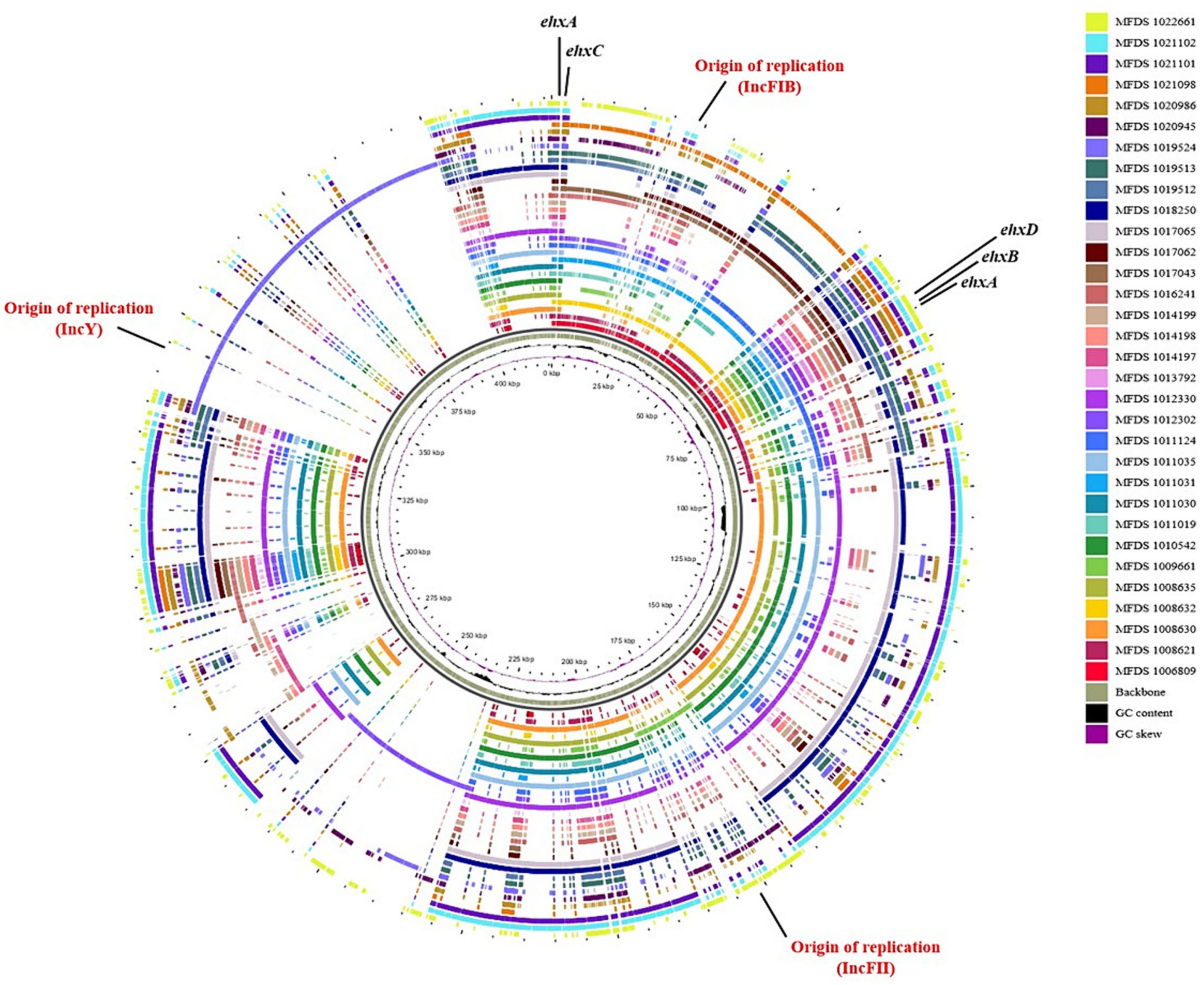
Comparative analysis of hemolysin-encoding plasmids. The pangenome shows the hemolysin-encoding plasmids for the 32 hybrid STEC/aEPEC strains. This pangenome is divided into plasmids IncFI1, IncFIB, IncY, and phage. The graphical circular maps of plasmids were generated using GView.

We conducted virulence gene mapping to identify an array of virulence factors within the STEC/aEPEC hybrid genome (Figure 1). All hybrid STEC/aEPEC strains harbored intimin (*eae*)- and Shiga toxin (*stx*)-encoding genes. In addition, numerous virulence determinants contributed to *E. coli* pathogenesis, including LEE-associated genes, enterotoxins, and additional components such as the LEE-locus-encoded type III secretion system (TTSS), ACE type VI secretion system (T6SS), and LEE-encoded TTSS effectors. Detailed results pertaining to virulence gene mapping of these hybrid genomes are documented in Supplementary Table S1. We also identified LEE-encoding genes associated with EPEC pathogenicity. Figure 2 presents a comparative analysis of 32 hybrid STEC/aEPEC strains, STEC strains (RM10042 (Parker et al., 2018) and RM10466 (Parker et al., 2018)), EPEC strains (E2348/69 (Iguchi et al., 2009) and E110019 (Hazen and Rasko, 2019)), and non-O157 EHEC (STEC/EPEC hybrid) strain (O26:H11 strains 11,368, O103:H2 strain 12,009). Genome data for each strain were acquired from the NCBI database, with the corresponding accession numbers as follows: RM10042 (CP028122), RM10466 (CP028381), E2348/69 (FM180568), E110019 (CP035751), 11,368 (AP010953), and 12,009 (AB253581). In a comparative assessment using EPEC strains E2348/69 and E110019 as positive controls, all hybrid STEC/aEPEC strains harbored the LEE region. Hemolysin plays a crucial role as a virulence factor in the prevalence *E. coli* UTIs and various extraintestinal locations (Elliott et al., 1998). Plasmid-carried enterohemolysin (*ehxA*) is a ubiquitous exotoxin produced by *E. coli* that amplifies the virulence of several clinical infections. To investigate the *ehxCABD* in hybrid STEC/aEPEC strains, we performed plasmid-related sequence analysis using PlasmidFinder 2.1. The analysis revealed the presence of three plasmid replicon sequences (IncFII, IncFIB, and IncY) associated with the established Inc. groups within the STEC/aEPEC genome, as indicated in Figure 3 and Supplementary Figure S1. Hemolysin-related genes were detected in all plasmids found in the 32 hybrid STEC/aEPEC strains (Figure 3).

### 3.4 *In silico* identification of antimicrobial resistance genes

**Figure 4:**
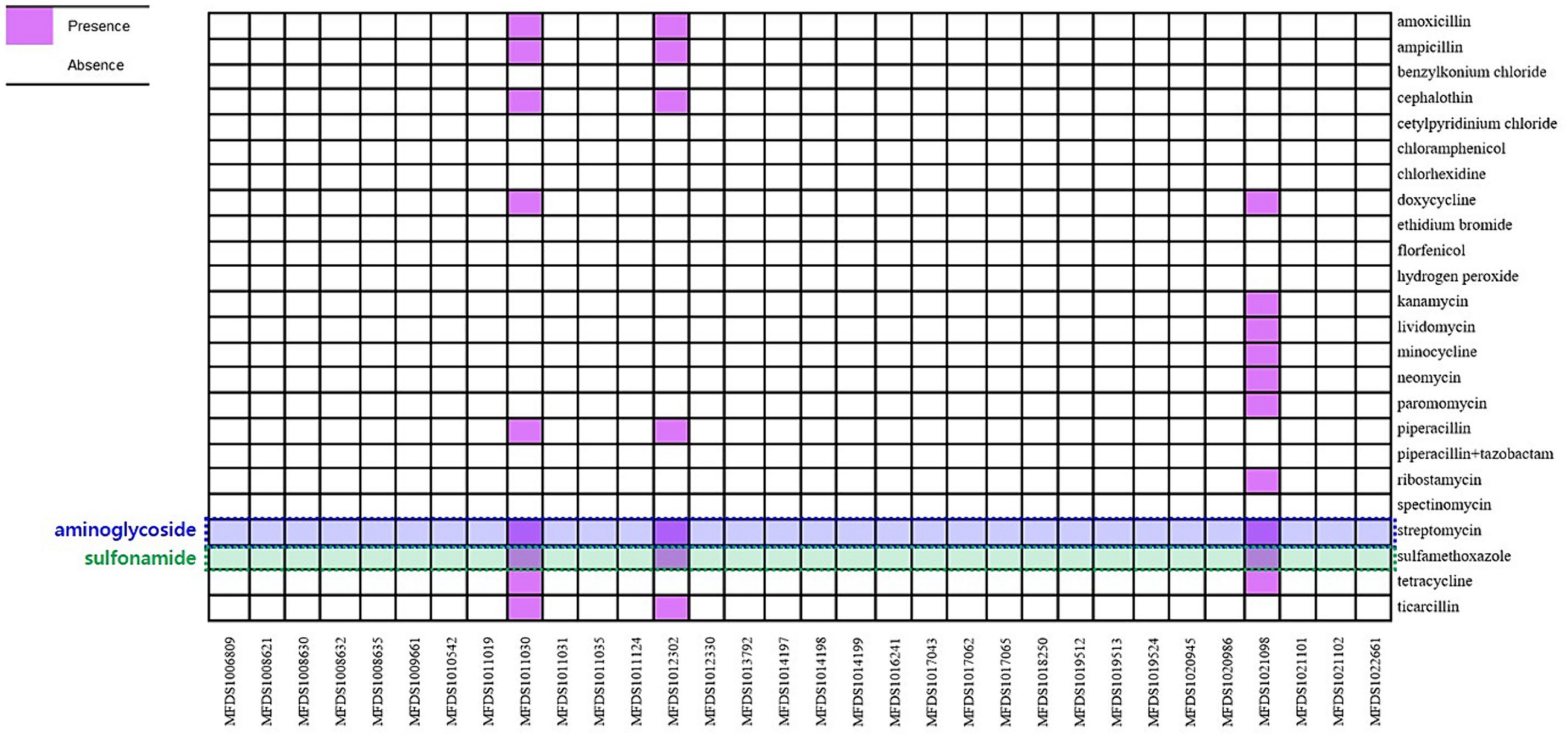
Heatmap of antimicrobial profiles across hybrid STEC/aEPEC strains. Heatmap showing the presence/absence of antimicrobial resistance (y-axis) among the hybrid STEC/aEPEC isolates identified in this study (x-axis). The presence of antimicrobial resistance genes is shown in purple, and the absence in white. Heatmap was generated using the gplot (v3.1.3) package in R software (v4.1.3).

ResFinder was used to predict the antimicrobial resistance genes within the hybrid genomes (Figure 4). Only three genomes (MFDS1011030, MFDS1012302, and MFDS1021098) harbored various antimicrobial resistance genes, with streptomycin- and sulfamethoxazole-related antibiotic resistance genes identified as common. Supplementary Table S2 shows the extensive mapping of antimicrobial resistance genes within hybrid *E. coli* genomes. MFDS1011030 harbored ampicillin resistance genes (*TEM-1B*), streptomycin resistance genes (*aph(3″)-Ib*, *aph(6)-Id*, *aph(3′)-la*), sulfamethoxazole resistance genes (*sul2*), and tetracycline resistance genes (*tet(A)*). MFDS1012302 harbored ampicillin resistance genes (*TEM-1B*) and streptomycin resistance genes (*aph(3″)-Ib*, *aph(6)-Id*), sulfamethoxazole resistance genes (*sul2*). MFDS1021098 streptomycin resistance genes (*aph(3″)-Ib*, *aph(6)-Id*, *aph(3′)-la*), sulfamethoxazole resistance genes (*sul2*), and tetracycline resistance genes (*tet(B)*). In particular, streptomycin resistance genes and sulfamethoxazole genes harbored common among the three STEC/aEPEC hybrids (MFDS1011030, MFDS1012302, and MFDS1021098).

### 3.5 Phage characterization

To detect the presence of *stx*-encoding phage in hybrid STEC/aEPEC strains, we conducted a comprehensive identification of bacteriophage sequences using PHASTER. The outcomes of our investigation, involving 32 hybrid STEC/aEPEC strains, are presented in Table 2. Validation of the entire set of *stx1* gene sequences was confirmed within the respective “intact” phage sequence region. Notably, the *stx1* (*stx 1a* and *stx 1b*) gene sequence was detected in most STEC/aEPEC strains (30/32), with only two strains (MFDS1009661 and MFDS1019524) confirmed to possess both *stx1* (*stx 1a* and *stx 1b*) and *stx2* (*stx 2a* and *stx 2b*) gene sequences.

### 3.6 Phylogenetic analysis and population structure analysis

**Figure 5:**
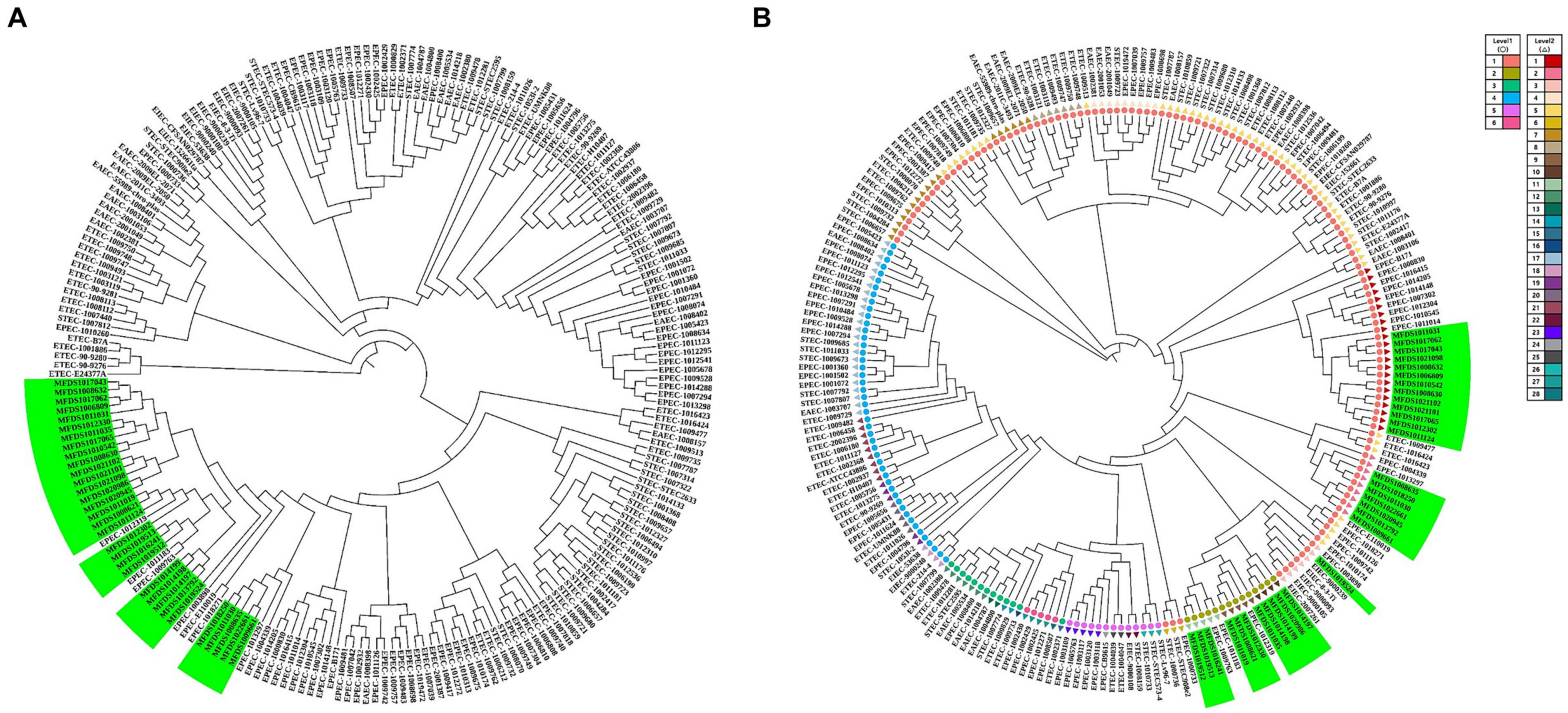
Phylogenetic and population structure analysis of hybrid STEC/aEPEC strains. **(A)** Green bars represent the 32 hybrid STEC/aEPEC strains that are closely related to specific EPEC strains. **(B)** Sequence clusters (1 to 6) are indicated in the outer colored dot, which are further divided into 28 lineages (inner ring). The green bar represents 32 STEC/EPEC hybrid strains isolated identified in this study. Among the identified strains, 32 hybrid strains closely related to EPEC were divided into six groups (two level 1, six level 2).

This study aimed to elucidate the genomic relationships between STEC/aEPEC hybrids and other pathogenic *E. coli* strains. The genomes of 187 isolates, comprising 41 STEC, 46 ETEC, 72 EPEC, 18 EAEC, and 10 EIEC strains, were used for the phylogenetic analysis to determine the genomic relationship between the STEC/aEPEC hybrids and other pathogenic *E. coli* isolates. The 187 genome datasets included the sequencing results of 160 pathogenic *E. coli* as well as 32 hybrid STEC/aEPEC genomes, which were deposited in the NCBI database. The genomes analyzed in this study are summarized in Supplementary Table S3. The phylogenetic analysis based on BPGA, which used coding sequences (CDSs) with protein sequences, revealed a close association between all hybrid strains and specific EPEC strains, suggesting the potential acquisition of STEC virulence genes during their emergence (Figure 5A). Furthermore, hierBAPS analysis was based on aligning SNPs from pan-genomes, and it’s utilized to identify genomic similarities among strains through hierarchical clustering of DNA sequence data. The phylogenetic analysis based on hierBAPS revealed their division into six primary sequence clusters (Bayesian analysis of population structure [BAPS] hierarchical level 1), which were further segregated into 28 lineages (BAPS level 2) (Figure 5B). All 32 hybrid strains were closely related to EPEC and were distributed across six groups (two at level 1 and six at level 2) within the total distribution.

### 3.7 Virulence of STEC/aEPEC hybrids

**Figure 6:**
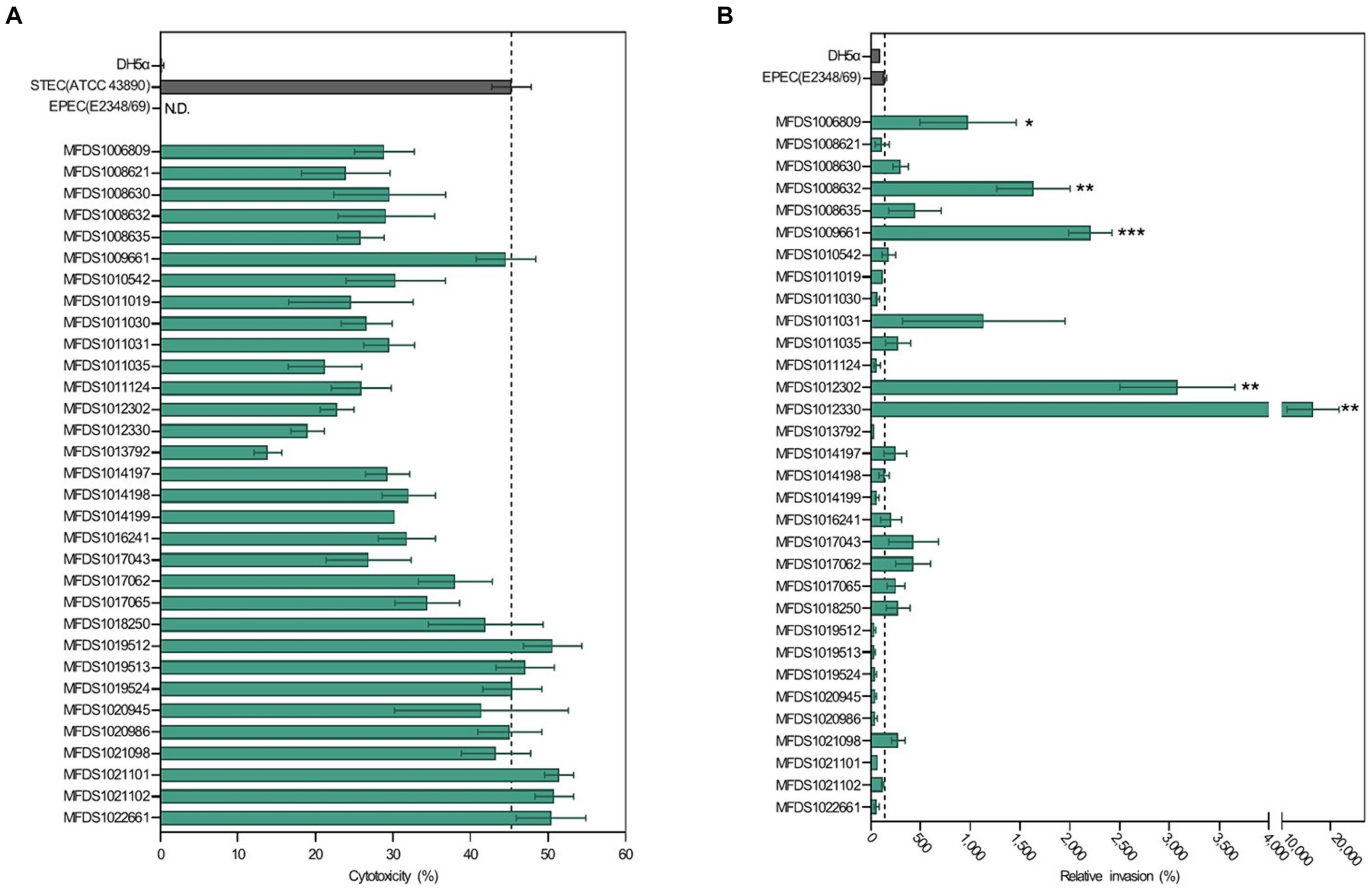
Assessment of cytotoxicity and invasive ability of STEC/aEPEC strains. **(A)** Cytotoxicity (%) of STEC/aEPEC hybrid strains was measured via LDH release from HeLa cells. Error bars represent S.E.M. from two independent experiments. ATCC 43890 and E2348_69 were used as reference strains for STEC and EPEC, respectively. N.D., Not detected. **(B)** The invasive ability of STEC/aEPEC hybrid strains was expressed as the relative invasion (%) of the negative control strain *E. coli* DH5α, defined as 100%. Error bars represent S.E.M. from two independent experiments. Statistical analyses were performed using Student’s *t*-test. *p < 0.05, **p < 0.01, ***p < =0.005.

As indicated in Figure 1, all STEC/aEPEC hybrids contained both *stx*- and LEE-encoding genes, suggesting the potential for enhanced virulence in a distinct set of pathotypes. Therefore, we evaluated the cytotoxic activities induced by Stx and the invasive abilities mediated by A/E formation in STEC/aEPEC hybrid strains (Figure 6). The cytotoxicity of the hybrid strains was determined by measuring the levels of cytoplasmic LDH released from HeLa cells. (Figure 6A). All hybrid strains and the STEC reference strain, ATCC 43890, were cytotoxic to HeLa cells (Maldonado et al., 2005), varying from 14% (MFDS1013792) to 51% (MFDS1021101). Among the 32 STEC/aEPEC hybrid strains, six (MFDS1019512, MFDS1019513, MFDS1019524, MFDS1022101, MFDS1021102, and MFDS1022661) exhibited higher cytotoxicity than the STEC strain ATCC 43890, although the differences were not statistically significant (*p* > 0.05, Student’s *t*-test). Notably, MFDS1009661 also exhibited high cytotoxic activity, similar to that of the STEC strain ATCC 43890.

The invasive abilities of hybrid STEC/aEPEC strains were also examined and compared to those of the EPEC strain E2348/69 (142%) and *E. coli* DH5α (100%), respectively (Figure 6B). Notably, five hybrid strains MFDS1006809 (979%, *p* = 0.0481), MFDS1008632 (1,635%, *p* = 0.0028), MFDS1009661 (2,207%, *p* = 0.0001), MFDS1012302 (3,080%, *p* = 0.0012), and MFDS1012330 (16,401%, *p* = 0.0078) exhibited significantly higher invasive ability than the control EPEC strain E2348/69 (142%). Moreover, MFDS1017062 and MFDS1021098 showed higher invasive abilities (428 and 280%, respectively) and a greater cytotoxicity potential (38 and 43%, respectively) than the control EPEC strain E2348/69 (142%).

## 4 Discussion

STEC and EPEC, both foodborne pathogens, can cause severe consequences such as potentially fatal infant diarrhea and hemolytic uremic syndrome, respectively. As STEC strains have the potential to induce diarrhea and hemolytic uremic syndrome (HUS) in human subjects regardless of the presence of LEE, this PAI is an accessory set of virulence genes that enhances STEC pathogenicity. The STEC/EPEC pathotype has been linked to numerous global outbreaks, severe symptoms, and, in some cases, fatal outcomes. Here, we first report 32 hybrid STEC/aEPEC strains identified in South Korea between 2015 and 2021, aiming to profile their virulence and associated genes for deeper exploration of their phylogenetic relationships with other pathogenic *E. coli* strains. Additionally, the genotypic profiles of AMR were analyzed. Furthermore, a cell-based assay was used to evaluate the virulence potential of hybrid STEC/aEPEC strains.

To molecularly characterize all hybrid STEC/aEPEC strains, a combination of real-time PCR and serotyping was used. WGS analysis of these hybrids corroborated the findings related to the presence of virulence factors, serotyping, and antimicrobial susceptibility. All 32 STEC/EPEC hybrid strains confirmed the presence of genes encoding Shiga toxin 1 and/or 2 (*stx1a*, *stx1b* and/or *stx2a*, *stx2b*) and *E. coli* attaching and effacing lesions (*eae*). Stxs, the major virulence factors of STEC, are broadly classified into two types: *stx1* and *stx2*. Furthermore, in the Stx1 group, stx1a is found in major STEC strains causing HC and HUS in humans, whereas *stx1c* and *stx1d* are rarely associated with human infections. In the Stx2 group, *stx2a*, *stx2c*, and *stx2d* were reported to be more virulent in human infections compared to isolates carrying *stx2b*, *stx2e*, *stx2f*, and *stx2g* (Burgos and Beutin, 2012; Probert et al., 2014; Zhi et al., 2019; Bai et al., 2021). Epidemiological data indicate a stronger association between Stx2-producing *E. coli* strains and HUS than strains producing Stx1 (Basu et al., 2016). However, the *E. coli* O157:H7 strain, producing *stx1*, and not *stx2*, was isolated from a Korean patient with HUS (Kim et al., 1998). Most hybrid STEC/aEPEC strains isolated from livestock feces, animal source foods, and water in South Korea harbored *stx1*. EPEC strains were categorized as typical or atypical based on the presence or absence of the *E. coli* adherence factor plasmid (EAF) housing *bfpA*, which encodes the bundle-forming pili (BFP). tEPEC includes the LEE region, including *eaeA* for attaching and effacing lesions, and *bfpA*. In contrast, aEPEC lacks *bfpA*. aEPEC causes endemic, epidemic, and persistent diarrhea in both humans and farm animals (Trabulsi et al., 2002), with certain cases linked to acute illness in humans (Abba et al., 2009). Our investigation confirmed that the STEC/EPEC hybrid strains identified in South Korea belonged to the aEPEC category and were distinguished by the absence of a gene encoding BFP.

All hybrid STEC/aEPEC strains encompassed chromosomally encoded virulence genes characteristic of EPEC, such as intimin and TTSS within the LEE region, and additional effectors not linked to LEE. The hallmark of an EPEC infection is the development of A/E lesions within the intestinal epithelium (Cepeda-Molero et al., 2017). These lesions result from genes situated on the LEE pathogenicity island, which encodes adhesin intimin, a TTSS, and six effectors, including the pivotal translocated intimin receptor (Tir) (Cepeda-Molero et al., 2017). In additionally, the chromosome of STEC/aEPEC contains T6SS which along with TTSS are important for cell–cell interaction and virulence factors. Previous studies have shown that the prevalence of the T6SS gene cluster is significantly higher in pathogenic *E. coli* strains compared to non-pathogenic *E. coli* strains. Pathogenic strains of *E. coli* have been identified with multiple T6SSs, some carrying more than one T6SS cluster, while others possess a single T6SS cluster but with multiple copies of *hcp* and *vgrG* genes. This highlights the functional role of the T6SS system in enhancing host pathogenicity.

The hybrid STEC/aEPEC strains in South Korea carry a phage-encoded STEC virulence gene (*stx*) responsible for Stx production. Additionally, phylogenetic tree analysis revealed close relationships between all hybrids and specific EPEC strains. These findings indicated that STEC/aEPEC hybrids potentially originated from EPEC strains that acquired STEC virulence genes via phage transmission. Moreover, our investigation revealed that all the hybrid strains possessed plasmid-carried enterohemolysin genes (*ehxCABD*). Enterohemolysin (*Ehx*) is a pore-forming toxin in enterohemorrhagic *Escherichia coli* that increases virulence in various clinical infections and associated with the presence of shiga toxin (Schwidder et al., 2019). This toxin is encoded on plasmids as part of an operon comprised of four genes: *ehxA* (coding for functional toxin), *ehxC* (managing post-translational modification of EhxA), *ehxB* and *ehxD* (responsible for the transport of EhxA through the inner membrane) (Kolenda et al., 2021).

The hybrids represented diverse serotypes [O103:H2 (*n* = 13), O26:H11 (*n* = 3), O74:H25 (*n* = 3), O84:H2 (*n* = 3), O108:H25 (*n* = 3), O98:H21 (*n* = 2), O103:H8 (*n* = 2), O71:H8 (*n* = 1), O156:H25 (*n* = 1), and O177:H25 (*n* = 1)]. The O157:H7 serotype strains are the primary human pathogens among enterohemorrhagic *E. coli* (EHEC) strains; however, other non-O157 serotypes (O26, O91, O103, O111, O121, and O145) have also exhibited the capacity to cause severe illnesses (Allerberger et al., 2003; Karmali et al., 2003; Kaper et al., 2004; Johnson et al., 2006; Messens et al., 2015; Soysal et al., 2016). Moreover, incidents of EHEC O80:H2 triggering lethal complications in HUS have recently emerged in France (Mariani-Kurkdjian et al., 2014; Soysal et al., 2016), the Netherlands (Wijnsma et al., 2017), and Switzerland (Nüesch-Inderbinen et al., 2018).

The cytotoxicity of Shiga toxin-producing *E. coli* is linked to the presence of *stx* (Maldonado et al., 2005). Polymyxin B is effective in extracting intracellular proteins, including heat-labile enterotoxins and Shiga-like toxins, from the periplasmic space of gram-negative bacteria (Evans et al., 1974; Karmali et al., 1985). Using the Polymyxin B extraction method for bacterial preparations, we examined the toxic potential of Stxs in STEC/aEPEC hybrids. All hybrid strains harboring *stx* and the STEC reference strain ATCC 43890 were cytotoxic to HeLa cells, whereas the EPEC reference strain E2348/69 and non-cytotoxic control *E. coli* DH5α showed no cytotoxicity. Notably, hybrid strains (MFDS1009661 and MFDS1019524) harboring both *stx1* and *stx2* were highly cytotoxic (45%) (Table 2), without discernible differences in cytotoxicity from the STEC strain ATCC 43890 (*p* > 0.05, Student’s *t*-test). Some hybrid strains lacking the *stx2* gene sequence also exhibited high cytotoxicity to HeLa cells, suggesting the involvement of other unidentified virulence genes in the cytotoxicity of hybrid STEC/aEPEC strains.

EPEC adherence occurs through three stages of adherence to epithelial cells: initial adherence (i), signal transduction (ii), and intimate attachment (iii) (Donnenberg et al., 1997). In particular, the EPEC adherence factor (EAF) plasmid confers localized adherence on the epithelial cells called the attaching and effacing (A/E) lesions by expressing BFP, which makes differentiation between tEPEC and aEPEC (Nataro and Kaper, 1998; Trabulsi et al., 2002). It is also distinct from the invasion mechanism of enteroinvasive *E. coli* (EIEC), which is mediated by the invasion plasmid (pINV) encoding genetic determinants for epithelial cell invasion, including the invasion plasmid antigen gene H (*ipaH*) (Sethabutr et al., 1993). The results of virulence gene mapping for 32 STEC/aEPEC strains demonstrated that *ipaH* sequences were not detected in the hybrid genomes (Figure 1). Considering the lack of EAF plasmid in aEPEC strains, the absence of *ipaH* suggests that the invasive potential of STEC/aEPEC hybrids may be derived from mechanisms different from those of tEPEC or EIEC. Instead, it was reported that some aEPEC strains possessing the intimin-encoding gene (*eae*) exhibited invasive potential in human intestinal cells *in vitro* with varying invasion efficiencies (Hernandes et al., 2008; Yamamoto et al., 2009). Despite the absence of the genes responsible for tEPEC and EIEC invasion, the presence of intimin within the genomes of 32 STEC/aEPEC hybrids may support the possibility of highly invasive strains. However, further analyses are still needed to evaluate the ability of hybrid strains to invade the epithelial cells *in vivo*. As the STEC strains are inefficient in invading epithelial cells (Oelschlaeger et al., 1994), invasive ability distinguishes EPEC from STEC types. The five hybrid strains were highly invasive to HeLa cells compared to the EPEC strain E2348/69. Notably, all five hybrid strains were related to EPEC in the phylogenetic analysis, suggesting a correlation between the phylogenetic and virulence profiles of the STEC/aEPEC hybrids. Moreover, all ST 1034 (*n* = 11) hybrid STEC/aEPEC strains displayed higher invasive potential than the EPEC strain E2348/69, including the highly invasive and cytotoxic hybrid strains MFDS1017062 and MFDS1021098. In particular, these genomes harbored two T6SS gene clusters, each containing the 13 core T6SS genes (TssJ, TssL, TssM, TssK, TssF, TssG, TssE, TssA, TssB, TssC, ClpV, Hcp, VgrG) necessary for the proper functioning of a T6SS. These results suggested the need to explore possible correlations between STs and virulence profiles in hybrid *E. coli* strains.

This study investigated the transfer of virulence genes in hybrid STEC/aEPEC strains identified in South Korea, emphasizing the efficacy of WGS as a robust method for scrutinizing bacterial genomes, particularly in detecting regions containing MGEs like phages and plasmids. Antibiotic resistance was confirmed in only three hybrid STEC/aEPEC strains (MFDS1011030, MFDS1012302, and MFDS1021098), displaying multiple drug resistance. Moreover, five hybrid strains (MFDS1006809, MFDS1008632, MFDS1009661, MFDS1012302, and MFDS1012330) showed cytotoxic activity against HeLa cells and significantly higher invasive ability than the EPEC strain E2348/69. These genomic and virulence findings substantially contribute to our understanding of hybrid STEC/aEPEC strains and pave the way for further investigations of *E. coli* pathogenicity. Furthermore, this study observes the emergence of MDR hybrid *E. coli* strains with high virulence potential.

## 5 Conclusion

This study documented the virulence markers and antimicrobial resistance profiles of hybrid STEC/aEPEC strains isolated in South Korea. Comprehensive genome-wide phylogenetic analysis demonstrated close genetic relationships between all hybrids and specific EPEC strains. Furthermore, the genes encoding *stx1* or *stx2* within these hybrid strains were harbored by phages. Additionally, some hybrid strains exhibited cytotoxicity and invasive abilities in human epithelial HeLa cells. Notably, all hybrid STEC/aEPEC strains that belonged to ST 1034 (*n* = 11) had a higher invasive potential than the EPEC strain E2348/69, including the two highly invasive and highly cytotoxic hybrid strains MFDS1017062 and MFDS1021098. Our results emphasize the potential increased danger to humans posed by hybrid STEC/aEPEC strains isolated in South Korea, containing both *stx* and *eaeA*, compared to STEC or EPEC alone. Furthermore, these genomic and virulence findings considerably contribute to our understanding of hybrid STEC/aEPEC strains and encourage further investigations into *E. coli* pathogenicity.

## Data availability statement

The original contributions presented in the study are included in the article/Supplementary material, further inquiries can be directed to the corresponding authors.

## Author contributions

WL: Data curation, Methodology, Writing – original draft. JH: Data curation, Methodology, Writing – review & editing. JC: Data curation, Methodology, Writing – review & editing. YJ: Data curation, Methodology, Writing – review & editing. EK: Methodology, Writing – review & editing. EA: Methodology, Writing – review & editing. SeK: Writing – review & editing. HS: Writing – review & editing. SR: Writing – review & editing. SoK: Conceptualization, Supervision, Writing – review & editing. H-YK: Supervision, Writing – review & editing.

## Footnotes

^1^https://github.com/rrwick/Unicycler

^2^https://cge.food.dtu.dk/services/MobileElementFinder/
