# Supplementary figures and images for: Genetic and virulence characteristics of hybrid Shiga toxin-producing and atypical enteropathogenic Escherichia coli strains isolated in South Korea
Source: Front Microbiol. 2024 May 15;15:1398262. doi: 10.3389/fmicb.2024.1398262 (PMC11133561; doi:10.3389/fmicb.2024.1398262)

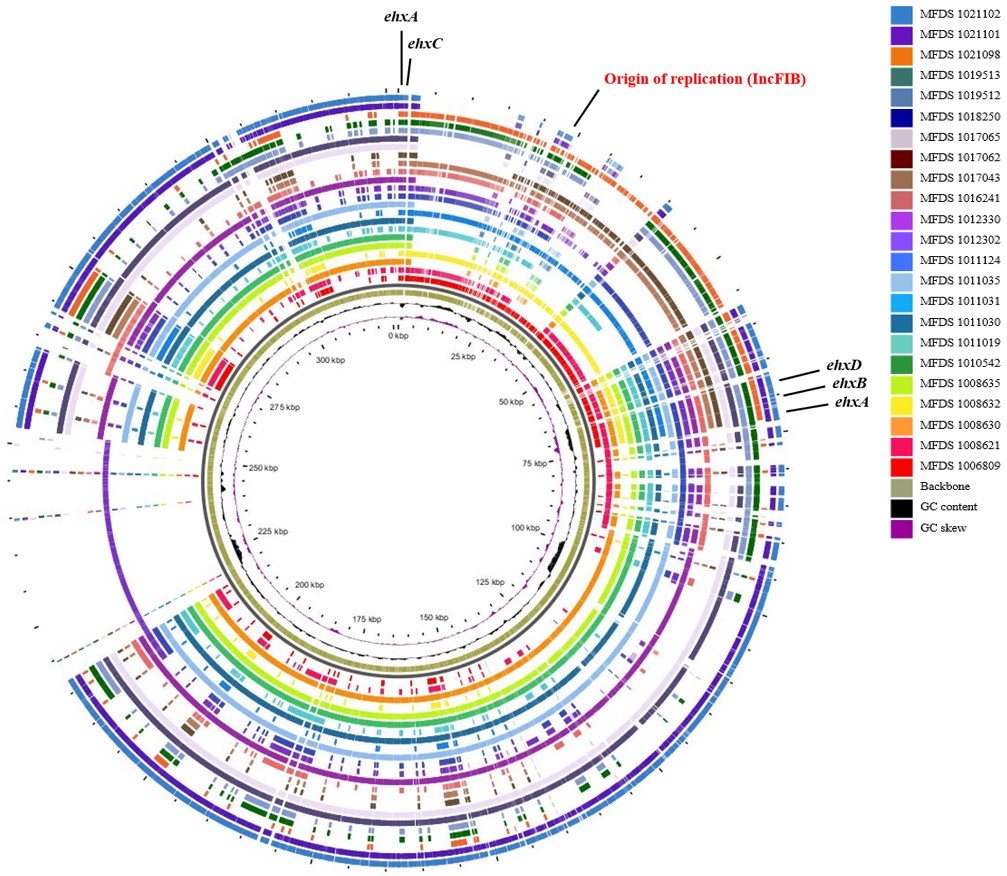

Supplement: Supplementary file 1 [file Image_1.JPEG]

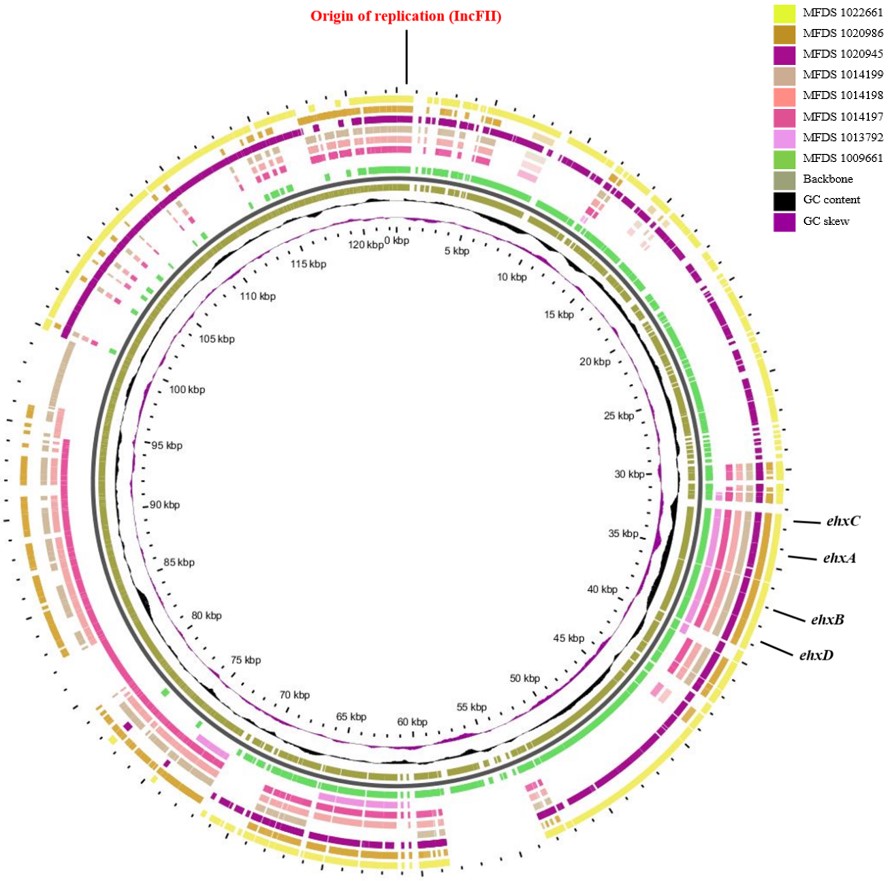

Supplement: Supplementary file 2 [file Image_2.JPEG]

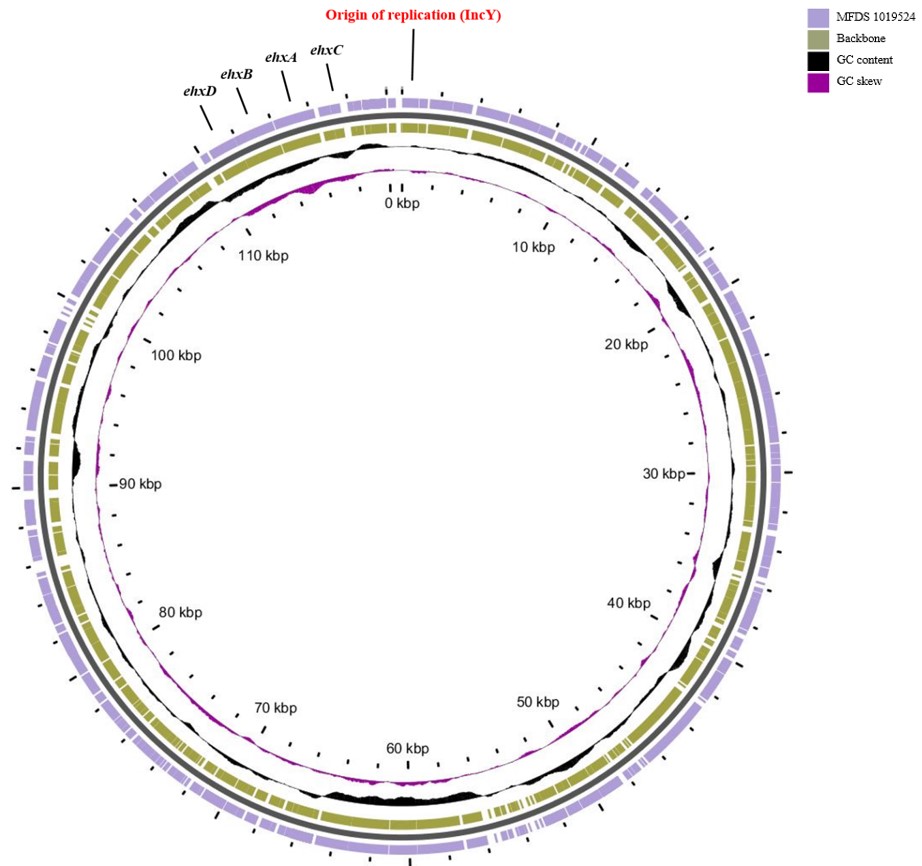

Supplement: Supplementary file 3 [file Image_3.JPEG]

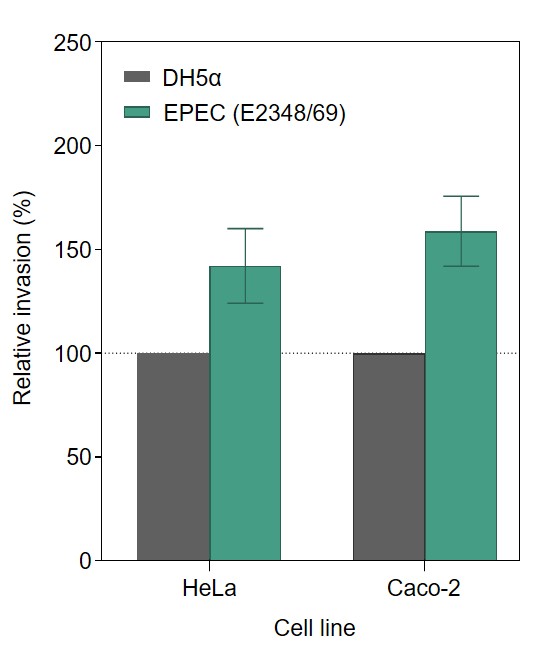

Supplement: Supplementary file 4 [file Image_4.JPEG]
