# Supplementary material for: Genetic and virulence characteristics of hybrid Shiga toxin-producing and atypical enteropathogenic Escherichia coli strains isolated in South Korea
Source: Front Microbiol. 2024 May 15;15:1398262. doi: 10.3389/fmicb.2024.1398262 (PMC11133561; doi:10.3389/fmicb.2024.1398262)
Supplement: Supplementary file 7 [file Table_3.DOCX]

**Table S3. Summarized characteristics of pathogenic *E.coli* strains**

| **Strain name** | **Collection date** | **Geographic location** | **Coverage** | **Contigs** | **Size (bp)** | **GC (%)** | **CDSs** | **rRNA** | **tRNA** | **Pathotype** | **Accession no.** |
| --- | --- | --- | --- | --- | --- | --- | --- | --- | --- | --- | --- |
| MFDS1000733 | 2012-06-28 | Incheon | 155 | 317 | 4,986,545 | 50.6 | 5113 | 8 | 77 | EPEC | SRR23851467 |
| MFDS1000736 | 2012-07-10 | Incheon | 86 | 293 | 5,217,997 | 50.7 | 5418 | 4 | 84 | STEC | SRR23851453 |
| MFDS1000829 | 2012-03-30 | Daejeon | 213 | 74 | 4,916,387 | 50.7 | 4813 | 3 | 77 | ETEC | SRR23851440 |
| MFDS1000830 | 2012-10-11 | Daejeon | 217 | 286 | 5,103,957 | 50.5 | 5292 | 8 | 84 | EPEC | SRR23851451 |
| MFDS1001072 | 2012-06-15 | Chungcheongbuk-do | 151 | 330 | 5,090,733 | 50.3 | 5289 | 7 | 80 | EPEC | SRR23851434 |
| MFDS1001360 | 2013-06-24 | Gyeongsangbuk-do | 250 | 187 | 5,049,991 | 50.3 | 5182 | 5 | 73 | EPEC | SRR23851423 |
| MFDS1001368 | 2013-06-26 | Gwangju | 82 | 271 | 5,049,628 | 50.7 | 5143 | 6 | 84 | STEC | SRR23851412 |
| MFDS1001502 | 2013-05-30 | Incheon | 214 | 206 | 5,018,758 | 50.4 | 5234 | 9 | 89 | EPEC | SRR23851411 |
| MFDS1001886 | 2013-09-30 | Busan | 55 | 175 | 4,994,824 | 50.5 | 5055 | 6 | 79 | ETEC | SRR23851410 |
| MFDS1002368 | 2013-12-04 | Gangwon-do | 93 | 478 | 5,071,920 | 50.5 | 5319 | 6 | 80 | ETEC | SRR23851409 |
| MFDS1002371 | 2013-12-04 | Gangwon-do | 86 | 541 | 5,242,780 | 50.7 | 5412 | 10 | 83 | ETEC | SRR23851454 |
| MFDS1002380 | 2013-12-04 | Gangwon-do | 80 | 1166 | 5,815,734 | 49.7 | 6508 | 10 | 86 | EAEC | SRR23851455 |
| MFDS1002381 | 2013-12-04 | Gangwon-do | 82 | 1255 | 5,604,760 | 49.8 | 6403 | 13 | 84 | EAEC | SRR23851456 |
| MFDS1002417 | 2013-12-17 | Gangwon-do | 89 | 884 | 5,569,704 | 50.1 | 6207 | 10 | 78 | STEC | SRR23851457 |
| MFDS1002425 | 2013-04-17 | Chungcheongnam-do | 303 | 60 | 4,692,424 | 50.6 | 4541 | 6 | 75 | EPEC | SRR23851458 |
| MFDS1002429 | 2013-04-09 | Chungcheongnam-do | 285 | 54 | 4,689,768 | 50.6 | 4544 | 4 | 71 | EPEC | SRR23851459 |
| MFDS1002430 | 2013-03-21 | Chungcheongnam-do | 282 | 115 | 4,707,905 | 50.6 | 4584 | 5 | 78 | EPEC | SRR23851460 |
| MFDS1002932 | 2013-08-14 | Jeollanam-do | 282 | 522 | 4,955,402 | 50.5 | 5168 | 5 | 79 | EPEC | SRR23851461 |
| MFDS1002937 | 2013-07-24 | Jeollanam-do | 276 | 154 | 4,709,289 | 50.9 | 4683 | 4 | 77 | ETEC | SRR23851462 |
| MFDS1003106 | 2013-06-28 | Incheon | 113 | 385 | 5,248,488 | 50.5 | 5452 | 6 | 85 | EAEC | SRR23851463 |
| MFDS1003109 | 2013-11-05 | Incheon | 177 | 176 | 5,035,365 | 50.6 | 4940 | 6 | 80 | EPEC | SRR23851441 |
| MFDS1003117 | 2013-11-12 | Incheon | 200 | 329 | 5,100,598 | 50.5 | 5112 | 6 | 84 | EPEC | SRR23851464 |
| MFDS1003118 | 2013-11-27 | Gyeonggi-do | 205 | 344 | 5,090,423 | 50.5 | 5105 | 6 | 83 | EPEC | SRR23851465 |
| MFDS1003119 | 2013-11-27 | Gyeonggi-do | 55 | 259 | 5,061,203 | 50.6 | 5243 | 8 | 82 | ETEC | SRR23851466 |
| MFDS1003120 | 2013-11-27 | Gyeonggi-do | 322 | 324 | 5,093,703 | 50.5 | 5092 | 12 | 80 | EPEC | SRR23851445 |
| MFDS1003121 | 2013-11-26 | Incheon | 55 | 837 | 5,351,232 | 49.9 | 5878 | 11 | 87 | ETEC | SRR23851446 |
| MFDS1003707 | 2014-04-22 | Incheon | 168 | 197 | 5,068,613 | 50.6 | 5166 | 4 | 88 | EAEC | SRR23851447 |
| MFDS1003898 | 2014-05-28 | Incheon | 258 | 228 | 5,183,006 | 50.5 | 5460 | 4 | 88 | EPEC | SRR23851448 |
| MFDS1004039 | 2014-06-16 | Jeollanam-do | 287 | 302 | 5,093,492 | 50.4 | 5184 | 9 | 84 | ETEC | SRR23851449 |
| MFDS1004047 | 2014-06-10 | Jeollanam-do | 269 | 217 | 5,014,495 | 50.4 | 5034 | 9 | 84 | ETEC | SRR23851450 |
| MFDS1004284 | 2014-02-03 | Gwangju | 181 | 297 | 5,262,232 | 50.7 | 5477 | 8 | 88 | STEC | SRR23851452 |
| MFDS1004339 | 2014-04-14 | Gwangju | 270 | 315 | 5,070,675 | 50.6 | 5303 | 8 | 83 | EPEC | SRR23851429 |
| MFDS1004787 | 2014-02-18 | Gangwon-do | 174 | 152 | 5,265,479 | 50.4 | 5218 | 9 | 86 | EAEC | SRR23851444 |
| MFDS1004796 | 2014-08-02 | Gangwon-do | 204 | 350 | 5,109,036 | 50.5 | 5305 | 6 | 87 | EPEC | SRR23851443 |
| MFDS1004800 | 2014-11-04 | Gangwon-do | 254 | 289 | 5,353,952 | 50.5 | 5427 | 10 | 94 | EAEC | SRR23851442 |
| MFDS1005423 | 2015-03-02 | Gwangju | 167 | 561 | 5,518,156 | 50.3 | 5993 | 6 | 79 | EPEC | SRR23851439 |
| MFDS1005431 | 2015-03-02 | Gwangju | 346 | 1268 | 5,602,139 | 50.1 | 6580 | 9 | 91 | EPEC | SRR23851438 |
| MFDS1005534 | 2015-04-22 | Ulsan | 189 | 216 | 5,255,454 | 50.6 | 5334 | 6 | 91 | EAEC | SRR23851437 |
| MFDS1005656 | 2015-04-27 | Gwangju | 172 | 370 | 5,387,389 | 50.4 | 5695 | 8 | 94 | EPEC | SRR23851436 |
| MFDS1005678 | 2015-05-25 | Gwangju | 175 | 314 | 5,212,397 | 50.5 | 5476 | 8 | 85 | EPEC | SRR23851435 |
| MFDS1005756 | 2015-08-25 | Gyeonggi-do | 160 | 279 | 4,889,956 | 50.7 | 4998 | 8 | 81 | ETEC | SRR23851433 |
| MFDS1005763 | 2015-08-28 | Gyeonggi-do | 152 | 170 | 5,038,664 | 50.6 | 4948 | 10 | 81 | EPEC | SRR23851432 |
| MFDS1006180 | 2015-10-26 | Incheon | 113 | 490 | 4,863,935 | 50.6 | 5158 | 6 | 79 | ETEC | SRR23851431 |
| MFDS1006199 | 2015-05-04 | Jeollanam-do | 218 | 343 | 5,105,114 | 50.7 | 5216 | 9 | 82 | STEC | SRR23851430 |
| MFDS1006212 | 2015-05-23 | Jeollanam-do | 241 | 453 | 5,009,353 | 50.6 | 5206 | 5 | 79 | ETEC | SRR23851408 |
| MFDS1006458 | 2015-08-19 | Chungcheongnam-do | 97 | 372 | 4,930,872 | 50.5 | 5137 | 12 | 65 | ETEC | SRR23851428 |
| MFDS1006494 | 2015-11-26 | Chungcheongnam-do | 177 | 373 | 5,464,751 | 50.7 | 5774 | 6 | 83 | STEC | SRR23851427 |
| MFDS1006657 | 2015-08-06 | Gwangju | 209 | 196 | 5,099,659 | 50.7 | 5177 | 4 | 90 | STEC | SRR23851426 |
| MFDS1006808 | 2015-06-09 | Jeollabuk-do | 207 | 1564 | 5,791,139 | 50.6 | 6966 | 9 | 96 | EPEC | SRR23851425 |
| MFDS1006810 | 2015-06-16 | Jeollabuk-do | 204 | 239 | 5,053,696 | 50.6 | 5183 | 9 | 87 | EPEC | SRR23851424 |
| MFDS1007039 | 2016-08-22 | Daegu | 133 | 238 | 4,903,481 | 50.7 | 4945 | 7 | 83 | EPEC | SRR23851422 |
| MFDS1007042 | 2016-09-02 | Chungcheongbuk-do | 225 | 156 | 4,779,554 | 50.6 | 4729 | 10 | 76 | EPEC | SRR23851421 |
| MFDS1007291 | 2016-05-17 | Jeollabuk-do | 214 | 462 | 5,141,778 | 50.4 | 5426 | 4 | 61 | EPEC | SRR23851420 |
| MFDS1007294 | 2016-05-24 | Jeollabuk-do | 225 | 911 | 5,340,735 | 50.5 | 5918 | 5 | 86 | EPEC | SRR23851419 |
| MFDS1007302 | 2016-07-04 | Gyeonggi-do | 226 | 255 | 5,199,726 | 50.6 | 5406 | 8 | 85 | EPEC | SRR23851418 |
| MFDS1007304 | 2016-07-04 | Gyeonggi-do | 194 | 147 | 4,972,292 | 50.6 | 4992 | 9 | 84 | EPEC | SRR23851417 |
| MFDS1007314 | 2016-05-30 | Gyeongsangbuk-do | 235 | 640 | 5,339,017 | 50.5 | 5699 | 8 | 82 | STEC | SRR23851416 |
| MFDS1007322 | 2016-07-18 | Gwangju | 177 | 314 | 4,969,455 | 50.7 | 5081 | 10 | 83 | STEC | SRR23851415 |
| MFDS1007440 | 2016-07-02 | Incheon | 151 | 475 | 5,027,796 | 50.5 | 5241 | 6 | 82 | ETEC | SRR23851414 |
| MFDS1007774 | 2016-05-09 | Jeollanam-do | 265 | 1212 | 5,114,715 | 50.6 | 5565 | 4 | 74 | STEC | SRR23851413 |
| MFDS1007787 | 2016-06-15 | Jeollanam-do | 226 | 435 | 4,689,229 | 50.8 | 4735 | 6 | 72 | STEC | SRR23867020 |
| MFDS1007792 | 2016-06-28 | Jeollanam-do | 232 | 737 | 5,413,787 | 50.6 | 5864 | 5 | 79 | STEC | SRR23867019 |
| MFDS1007799 | 2016-07-25 | Jeollanam-do | 292 | 598 | 4,988,758 | 51 | 5286 | 3 | 76 | STEC | SRR23867008 |
| MFDS1007807 | 2016-08-23 | Jeollanam-do | 225 | 1444 | 5,263,382 | 50.8 | 5924 | 4 | 68 | STEC | SRR23866997 |
| MFDS1007812 | 2016-08-30 | Jeollanam-do | 220 | 1162 | 5,309,919 | 50.8 | 6003 | 5 | 75 | STEC | SRR23866986 |
| MFDS1007818 | 2016-09-26 | Jeollanam-do | 212 | 464 | 5,071,985 | 50.6 | 5130 | 4 | 78 | EPEC | SRR23866975 |
| MFDS1008070 | 2016-11-22 | Daejeon | 250 | 891 | 5,049,444 | 50.8 | 5460 | 5 | 74 | STEC | SRR23866964 |
| MFDS1008074 | 2016-11-30 | Daejeon | 214 | 2793 | 5,945,913 | 49.5 | 7771 | 9 | 118 | EPEC | SRR23866963 |
| MFDS1008112 | 2016-08-22 | Seoul | 135 | 146 | 5,032,981 | 50.6 | 5061 | 6 | 80 | ETEC | SRR23866962 |
| MFDS1008113 | 2016-08-22 | Seoul | 53 | 1306 | 6,476,170 | 50.6 | 7473 | 4 | 89 | ETEC | SRR23866961 |
| MFDS1008157 | 2016-03-21 | Ulsan | 234 | 1105 | 5,002,886 | 50.8 | 5427 | 6 | 66 | EAEC | SRR23867018 |
| MFDS1008159 | 2016-03-03 | Ulsan | 189 | 2039 | 4,500,504 | 51.2 | 5834 | 6 | 57 | STEC | SRR23867017 |
| MFDS1008398 | 2016-04-29 | Chungcheongnam-do | 193 | 438 | 5,093,047 | 50.5 | 5309 | 10 | 84 | EAEC | SRR23867016 |
| MFDS1008400 | 2016-06-20 | Chungcheongnam-do | 229 | 1047 | 5,689,783 | 50.3 | 6327 | 9 | 87 | EPEC | SRR23867015 |
| MFDS1008401 | 2016-08-22 | Chungcheongnam-do | 169 | 382 | 5,169,262 | 50.5 | 5322 | 8 | 82 | EAEC | SRR23867014 |
| MFDS1008402 | 2016-08-22 | Chungcheongnam-do | 209 | 473 | 5,198,164 | 50.5 | 5522 | 5 | 78 | EAEC | SRR23867013 |
| MFDS1008408 | 2016-11-22 | Chungcheongnam-do | 227 | 304 | 5,160,252 | 50.6 | 5316 | 8 | 81 | STEC | SRR23867012 |
| MFDS1008507 | 2017-06-15 | Jeollanam-do | 108 | 474 | 5,108,471 | 50.6 | 5385 | 7 | 81 | EPEC | SRR23867011 |
| MFDS1008634 | 2017-05-22 | Jeollabuk-do | 113 | 423 | 5,551,194 | 50.3 | 5990 | 7 | 82 | EPEC | SRR23867010 |
| MFDS1008698 | 2017-04-13 | Gyeongsangbuk-do | 303 | 269 | 4,948,421 | 50.6 | 5035 | 6 | 82 | EPEC | SRR23867009 |
| MFDS1009417 | 2017-09-20 | Gyeongsangbuk-do | 253 | 247 | 4,992,953 | 50.6 | 5130 | 5 | 78 | EPEC | SRR23867007 |
| MFDS1009477 | 2016-10-13 | Incheon | 243 | 222 | 5,194,533 | 50.4 | 5315 | 7 | 78 | ETEC | SRR23867006 |
| MFDS1009478 | 2016-10-18 | Incheon | 185 | 161 | 4,956,666 | 50.7 | 4839 | 7 | 82 | ETEC | SRR23867005 |
| MFDS1009481 | 2016-10-18 | Incheon | 270 | 446 | 5,152,489 | 50.3 | 5377 | 8 | 76 | EPEC | SRR23867004 |
| MFDS1009482 | 2016-11-14 | Incheon | 187 | 385 | 5,159,579 | 50.5 | 5410 | 7 | 81 | ETEC | SRR23867003 |
| MFDS1009483 | 2017-04-25 | Incheon | 244 | 707 | 4,986,125 | 50.6 | 5292 | 6 | 70 | EPEC | SRR23867002 |
| MFDS1009493 | 2017-06-26 | Incheon | 180 | 244 | 4,922,029 | 50.6 | 4982 | 4 | 81 | ETEC | SRR23867001 |
| MFDS1009513 | 2017-08-28 | Incheon | 171 | 240 | 5,105,434 | 50.5 | 5212 | 6 | 84 | ETEC | SRR23867000 |
| MFDS1009528 | 2017-07-17 | Chungcheongbuk-do | 179 | 234 | 5,309,329 | 50.4 | 5545 | 5 | 83 | EPEC | SRR23866999 |
| MFDS1009657 | 2017-07-17 | Jeollabuk-do | 184 | 123 | 5,026,485 | 50.8 | 5017 | 6 | 85 | STEC | SRR23866996 |
| MFDS1009673 | 2017-09-12 | Jeollabuk-do | 185 | 280 | 5,259,307 | 50.4 | 5451 | 9 | 84 | STEC | SRR23866995 |
| MFDS1009675 | 2017-09-18 | Jeollabuk-do | 270 | 294 | 5,070,912 | 50.5 | 5233 | 4 | 80 | EPEC | SRR23866994 |
| MFDS1009680 | 2017-10-11 | Jeollabuk-do | 230 | 142 | 5,217,763 | 50.5 | 5325 | 4 | 79 | STEC | SRR23866993 |
| MFDS1009685 | 2017-10-24 | Jeollabuk-do | 229 | 404 | 5,260,771 | 50.5 | 5501 | 8 | 81 | STEC | SRR23866992 |
| MFDS1009721 | 2016-09-20 | Incheon | 207 | 1500 | 4,818,016 | 50.7 | 5440 | 5 | 67 | STEC | SRR23866991 |
| MFDS1009723 | 2016-09-20 | Incheon | 236 | 250 | 5,183,956 | 50.7 | 5397 | 4 | 79 | STEC | SRR23866990 |
| MFDS1009729 | 2017-06-29 | Incheon | 170 | 265 | 5,132,845 | 50.4 | 5307 | 7 | 77 | ETEC | SRR23866989 |
| MFDS1009732 | 2017-08-31 | Incheon | 231 | 291 | 5,079,029 | 50.7 | 5242 | 4 | 87 | STEC | SRR23866988 |
| MFDS1009733 | 2017-08-31 | Incheon | 241 | 177 | 5,450,231 | 50.2 | 5559 | 4 | 81 | ETEC | SRR23866987 |
| MFDS1009735 | 2017-08-31 | Incheon | 206 | 103 | 4,783,229 | 50.6 | 4715 | 4 | 71 | ETEC | SRR23866985 |
| MFDS1009740 | 2017-08-31 | Incheon | 239 | 92 | 4,859,418 | 50.7 | 4812 | 5 | 73 | ETEC | SRR23866984 |
| MFDS1009742 | 2017-08-31 | Incheon | 253 | 205 | 5,093,717 | 50.6 | 5283 | 4 | 75 | EPEC | SRR23866983 |
| MFDS1009747 | 2017-08-31 | Incheon | 229 | 1056 | 4,926,639 | 50.6 | 5281 | 4 | 58 | ETEC | SRR23866982 |
| MFDS1009748 | 2017-08-31 | Incheon | 221 | 486 | 4,913,650 | 50.7 | 5055 | 4 | 76 | ETEC | SRR23866981 |
| MFDS1009749 | 2017-08-31 | Incheon | 258 | 133 | 4,925,276 | 50.7 | 4952 | 4 | 77 | EPEC | SRR23866980 |
| MFDS1009750 | 2017-08-31 | Incheon | 201 | 1622 | 5,081,024 | 50.8 | 5919 | 3 | 67 | ETEC | SRR23866979 |
| MFDS1009757 | 2017-09-08 | Incheon | 178 | 187 | 5,047,310 | 50.5 | 5121 | 7 | 79 | EPEC | SRR23866978 |
| MFDS1009762 | 2017-05-20 | Incheon | 221 | 528 | 4,924,469 | 50.7 | 5050 | 5 | 71 | ETEC | SRR23866977 |
| MFDS1009763 | 2017-05-20 | Incheon | 258 | 362 | 5,224,631 | 50.6 | 5544 | 6 | 80 | EPEC | SRR23866976 |
| MFDS1010174 | 2017-08-18 | Gyeongsangnam-do | 237 | 2033 | 6,114,130 | 50.3 | 7819 | 5 | 88 | EPEC | SRR23866973 |
| MFDS1010260 | 2017-10-27 | Gyeongsangbuk-do | 225 | 2866 | 6,204,399 | 50.6 | 8354 | 5 | 83 | EPEC | SRR23866972 |
| MFDS1010313 | 2017-08-31 | Chungcheongnam-do | 216 | 599 | 4,980,461 | 50.6 | 5169 | 5 | 75 | EPEC | SRR23866971 |
| MFDS1010484 | 2017-05-23 | Gwangju | 180 | 513 | 5,339,897 | 50.4 | 5780 | 7 | 83 | EPEC | SRR23866970 |
| MFDS1010545 | 2017-08-28 | Jeollabuk-do | 181 | 290 | 5,388,409 | 50.3 | 5642 | 9 | 85 | EPEC | SRR23866969 |
| MFDS1010733 | 2017-12-04 | Jeollabuk-do | 231 | 313 | 5,192,798 | 50.6 | 5374 | 5 | 83 | STEC | SRR23866968 |
| MFDS1010859 | 2017-12-12 | Incheon | 174 | 1568 | 5,041,678 | 50.6 | 5938 | 7 | 68 | STEC | SRR23866967 |
| MFDS1010997 | 2018-02-06 | Jeollabuk-do | 241 | 1563 | 5,060,570 | 50.7 | 5911 | 4 | 65 | STEC | SRR23866965 |
| MFDS1011014 | 2018-03-12 | Jeollabuk-do | 348 | 335 | 5,404,611 | 50.5 | 5707 | 4 | 83 | EPEC | SRR23867158 |
| MFDS1011026 | 2018-04-23 | Jeollabuk-do | 278 | 339 | 5,512,231 | 50.3 | 5775 | 7 | 80 | ETEC | SRR23867157 |
| MFDS1011033 | 2018-05-08 | Jeollabuk-do | 211 | 252 | 5,318,095 | 50.6 | 5560 | 5 | 79 | STEC | SRR23867146 |
| MFDS1011123 | 2018-05-18 | Jeollanam-do | 319 | 376 | 5,375,775 | 50.4 | 5690 | 3 | 80 | EPEC | SRR23867135 |
| MFDS1011126 | 2018-05-18 | Incheon | 272 | 239 | 4,983,492 | 50.5 | 5098 | 6 | 69 | EPEC | SRR23867124 |
| MFDS1011127 | 2018-06-19 | Incheon | 257 | 232 | 4,964,708 | 50.7 | 5098 | 6 | 80 | ETEC | SRR23867120 |
| MFDS1011176 | 2018-06-04 | Jeollabuk-do | 269 | 206 | 5,314,655 | 50.7 | 5406 | 6 | 77 | STEC | SRR23867119 |
| MFDS1011181 | 2018-06-18 | Jeollabuk-do | 217 | 1836 | 6,373,314 | 47.4 | 7818 | 10 | 106 | STEC | SRR23867118 |
| MFDS1011183 | 2018-06-18 | Jeollabuk-do | 248 | 295 | 5,232,979 | 50.4 | 5545 | 5 | 83 | EPEC | SRR23867117 |
| MFDS1011624 | 2018-04-30 | Chungcheongbuk-do | 283 | 883 | 5,883,953 | 50.4 | 6578 | 6 | 91 | EPEC | SRR23867116 |
| MFDS1012271 | 2018-07-13 | Gyeongsangbuk-do | 278 | 105 | 4,786,924 | 50.7 | 4697 | 3 | 68 | EPEC | SRR23867156 |
| MFDS1012272 | 2018-09-10 | Ulsan | 194 | 490 | 5,202,824 | 50.4 | 5552 | 3 | 80 | EPEC | SRR23867155 |
| MFDS1012281 | 2018-08-29 | Seoul | 249 | 337 | 5,343,417 | 50.7 | 5447 | 4 | 76 | ETEC | SRR23867154 |
| MFDS1012295 | 2018-10-24 | Gyeongsangbuk-do | 260 | 340 | 5,476,050 | 50.3 | 5820 | 5 | 92 | EPEC | SRR23867153 |
| MFDS1012304 | 2018-07-24 | Jeollabuk-do | 251 | 258 | 5,335,178 | 50.4 | 5555 | 3 | 79 | EPEC | SRR23867152 |
| MFDS1012310 | 2018-08-21 | Jeollabuk-do | 237 | 257 | 5,365,029 | 50.8 | 5562 | 4 | 77 | STEC | SRR23867151 |
| MFDS1012319 | 2018-09-10 | Jeollabuk-do | 255 | 665 | 5,411,102 | 50.4 | 5958 | 4 | 81 | EPEC | SRR23867150 |
| MFDS1012327 | 2018-10-01 | Jeollabuk-do | 250 | 191 | 4,987,158 | 50.8 | 5003 | 4 | 71 | STEC | SRR23867149 |
| MFDS1012536 | 2018-07-23 | Chungcheongnam-do | 266 | 892 | 5,366,853 | 50.6 | 5887 | 8 | 72 | STEC | SRR23867148 |
| MFDS1012541 | 2018-10-26 | Chungcheongbuk-do | 233 | 325 | 5,453,131 | 50.3 | 5797 | 6 | 89 | EPEC | SRR23867147 |
| MFDS1013275 | 2019-05-21 | Gyeongsangbuk-do | 78 | 165 | 4,837,872 | 50.7 | 4869 | 7 | 81 | ETEC | SRR23867145 |
| MFDS1013297 | 2019-06-21 | Daegu | 128 | 970 | 5,348,510 | 49.6 | 6024 | 11 | 88 | EPEC | SRR23867144 |
| MFDS1013298 | 2019-01-31 | Seoul | 129 | 1325 | 5,875,999 | 48.9 | 6993 | 8 | 91 | EPEC | SRR23867143 |
| MFDS1014133 | 2019-03-11 | Gwangju | 103 | 1413 | 5,942,014 | 50.4 | 7011 | 7 | 84 | STEC | SRR23867142 |
| MFDS1014148 | 2019-09-19 | Gwangju | 129 | 1438 | 5,692,085 | 49.1 | 6776 | 10 | 88 | EPEC | SRR23867141 |
| MFDS1014205 | 2019-06-23 | Daejeon | 154 | 342 | 5,098,967 | 50.5 | 5294 | 11 | 89 | EPEC | SRR23867140 |
| MFDS1014218 | 2019-12-31 | Gyeongsangnam-do | 145 | 274 | 5,370,709 | 50.5 | 5502 | 9 | 84 | EAEC | SRR23867139 |
| MFDS1014288 | 2019-04-08 | Chungcheongbuk-do | 200 | 269 | 5,364,401 | 50.4 | 5639 | 7 | 90 | EPEC | SRR23867138 |
| MFDS1016415 | 2020-07-18 | Ulsan | 157 | 312 | 5,180,935 | 50.5 | 5423 | 10 | 89 | EPEC | SRR23867137 |
| MFDS1016423 | 2020-07-27 | Gyeongsangbuk-do | 106 | 721 | 5,029,473 | 50.1 | 5430 | 8 | 84 | ETEC | SRR23867136 |
| MFDS1016424 | 2020-07-30 | Gyeongsangbuk-do | 101 | 737 | 5,041,249 | 50 | 5474 | 9 | 78 | ETEC | SRR23867134 |
| MFDS1018271 | 2020-06-26 | Chungcheongnam-do | 117 | 353 | 5,019,718 | 50.5 | 5215 | 7 | 87 | EPEC | SRR23867133 |
| MFDS1019472 | 2021-07-27 | Jeollabuk-do | 121 | 269 | 4,960,555 | 50.6 | 5073 | 7 | 86 | EPEC | SRR23867131 |
| MFDS2001049 | 2016-06-17 | Jeju-do | 116 | 340 | 5,477,558 | 50.7 | 5762 | 5 | 84 | EAEC | SRR23867130 |
| MFDS2001053 | 2016-06-17 | Jeju-do | 81 | 281 | 5,388,694 | 50.6 | 5617 | 7 | 82 | EAEC | SRR23867129 |
| MFDS2001387 | 2017-05-18 | Chungcheongbuk-do | 165 | 210 | 5,001,955 | 50.6 | 5075 | 8 | 79 | EPEC | SRR23867128 |
| MFDS2002396 | 2015-02-01 | Busan |  | 5 | 5,100,852 | 50.6 | 5139 | 22 | 86 | ETEC | GCA_001750845.1 |
| MFDS2007261 | 2020-04-13 | Missing | 328 | 413 | 4,922,104 | 50.7 | 5206 | 5 | 75 | EIEC | SRR23867127 |
| MFDS9000093 | 2021-08-10 | Missing | 171 | 595 | 4,991,175 | 50.7 | 5553 | 5 | 85 | EIEC | SRR23867126 |
| MFDS9000105 | 2021-08-10 | Busan | 178 | 617 | 5,032,528 | 50.7 | 5554 | 5 | 82 | EIEC | SRR23867125 |
| MFDS9000108 | 2021-08-10 | Missing | 152 | 416 | 5,062,915 | 50.5 | 5320 | 8 | 89 | EIEC | SRR23867123 |
| MFDS9000239 | 2021-08-10 | Missing | 140 | 536 | 4,680,835 | 50.7 | 5083 | 5 | 78 | EIEC | SRR23867122 |
| MFDS9000240 | 2021-08-10 | Missing | 151 | 883 | 5,253,593 | 50.8 | 5820 | 7 | 91 | EIEC | SRR23867121 |
| 2009EL-2050 | Missing | Missing | - | 1 | 5,253,138 | 50.7 | 5220 | 22 | 94 | EAEC | GCA_000299255.1 |
| 2009EL-2071 | Missing | Missing | - | 1 | 5,312,586 | 50.7 | 5297 | 22 | 98 | EAEC | GCA_000299475.1 |
| 2011EL-3493 | Missing | Missing | - | 1 | 5,273,097 | 50.7 | 5251 | 22 | 94 | EAEC | GCA_000299455.1 |
| 55989 | Missing | Missing | - | 2 | 5,227,344 | 50.6 | 5201 | 22 | 94 | EAEC | GCA_000026245.1 |
| 8_3_Ti3 | 2010 | Australia | - | 3 | 5,141,085 | 50.8 | 5337 | 22 | 97 | EIEC | GCA_012221565.1 |
| 53638 | Missing | Missing | - | 4 | 5,371,790 | 51 | 5549 | 22 | 102 | EIEC | GCA_000167915.2 |
| 152661 | 2014-07 | United Kingdom | - | 3 | 5,199,422 | 50.6 | 7391 | 22 | 83 | EIEC | GCA_009762515.1 |
| CFSAN029787 | 2012-04-15 | Italy | - | 3 | 5,288,947 | 50.5 | 5381 | 22 | 104 | EIEC | GCA_001007915.1 |
| B171 | Missing | Missing | - | 265 | 5,654,428 | 50.5 | 6124 | 19 | 76 | EPEC | GCA_000167895.3 |
| CB9615 | Missing | Germany | - | 1 | 5,386,352 | 50.5 | 5325 | 22 | 100 | EPEC | GCA_000025165.1 |
| E110019 | Missing | Missing | - | 1 | 5,249,232 | 50.8 | 5311 | 22 | 92 | EPEC | GCA_000167875.1 |
| 90-9269 | 1988 | Bangladesh | - | 1 | 4,760,041 | 50.7 | 4731 | 22 | 89 | ETEC | GCA_002764155.1 |
| 90-9276 | 1988 | Bangladesh | - | 1 | 5,004,671 | 50.7 | 4983 | 22 | 91 | ETEC | GCA_002741235.1 |
| 90-9280 | 1988 | Bangladesh | - | 1 | 4,966,338 | 50.7 | 4880 | 22 | 91 | ETEC | GCA_002741255.1 |
| 90-9281 | 1988 | Bangladesh | - | 1 | 4,978,613 | 50.6 | 4881 | 22 | 89 | ETEC | GCA_002741455.1 |
| 214-4 | 1974 | Mexico | - | 1 | 5,138,709 | 50.7 | 5173 | 22 | 91 | ETEC | GCA_003956245.1 |
| ATCC-43886 | Missing | Missing | - | 1 | 4,914,654 | 50.8 | 4896 | 22 | 96 | ETEC | GCA_002741295.1 |
| B7A | Missing | Missing | - | 289 | 5,300,242 | 50.7 | 5510 | 17 | 65 | ETEC | GCA_000167815.1 |
| E24377A | Missing | Missing | - | 1 | 4,979,619 | 50.6 | 4875 | 22 | 91 | ETEC | GCA_000017745.1 |
| H10407 | Missing | Missing | - | 1 | 5,153,435 | 50.8 | 5147 | 22 | 90 | ETEC | GCA_000210475.1 |
| UMNK88 | Missing | USA | - | 1 | 5,186,416 | 50.7 | 5255 | 22 | 96 | ETEC | GCA_000212715.2 |
| STEC2633 | 2013-10 | Netherlands | - | 132 | 5,130,174 | 50.8 | 5202 | 4 | 76 | STEC | GCA_001607605.1 |
| STEC2595 | 2013-09 | Netherlands | - | 104 | 5,304,448 | 50.4 | 5334 | 6 | 82 | STEC | GCA_001607595.1 |
| STEC573-4 | 2016 | Netherlands | - | 128 | 5,214,238 | 50.4 | 5299 | 4 | 83 | STEC | GCA_002286585.1 |
| STEC908e2 | 2019-12-02 | China | - | 536 | 5,108,633 | 50.6 | 5406 | 2 | 93 | STEC | GCA_021504475.1 |
| STEC Lv96-7 | 2019-07-13 | China | - | 36 | 4,632,475 | 50.6 | 4475 | 8 | 75 | STEC | GCA_021504255.1 |
| STEC1053l-2 | 2020-03-13 | China | - | 468 | 5,303,606 | 50.4 | 5603 | 4 | 104 | STEC | GCA_021504535.1 |

S, coding sequence
